# A Dynamic DNA Nano‐Antioxidant Targeting Galectin‐3 Attenuates Liver Fibrosis via Reducing Macrophage Oxidative Stress

**DOI:** 10.1002/advs.202509977

**Published:** 2025-10-30

**Authors:** Mengjia Peng, Jianguo Xu, Youjian Hong, Fei Fang, Yan Li, Duo Ci, Bowen Wang

**Affiliations:** ^1^ Department of Emergency the General Hospital of Tibet Military Command Lhasa China; ^2^ Department of Gastroenterology Laboratory of Gastroenterology and Hepatology West China Hospital Sichuan University Chengdu China; ^3^ State Key Laboratory of Oral Diseases National Center for Stomatology National Clinical Research Center for Oral Diseases West China Hospital of Stomatology Sichuan University Chengdu China; ^4^ Physical Examination Center General Hospital of Western Theater Command Chengdu China

**Keywords:** dynamic DNA nano‐antioxidant, galectin‐3, liver fibrosis, NRF2, reactive oxygen species

## Abstract

Liver cirrhosis represents a major global health challenge with significant socioeconomic implications. Oxidative stress‐mediated injury plays a pivotal role in driving fibrotic progression, with hepatic macrophages serving as a dominant source of reactive oxygen species (ROS). Consequently, targeted suppression of macrophage‐derived ROS presents a promising therapeutic strategy. Single‐cell RNA sequencing analysis identified Galectin‐3 as positively correlated with macrophage oxidative stress and ROS production. Here, a dynamic DNA nano‐antioxidant (DDN) is developed for hepatic macrophage‐specific small interfering RNA (siRNA) delivery targeting Galectin‐3. DDN administration achieved a 3.92‐fold reduction in Galectin‐3 expression, with transcriptomic profiling revealing restoration of Nuclear Factor Erythroid 2‐Related Factor 2 (NRF2) signaling and consequent attenuation of oxidative stress in macrophages. Pharmacokinetic assessment via IVIS imaging demonstrated superior hepatic accumulation of DDN, exhibiting 1.57‐ and 2.11‐fold greater fluorescence intensity at 240 min post‐injection compared to siRNA and tetrahedral framework nucleic acid (tFNA), respectively. In a carbon tetrachloride (CCl_4_)‐induced mouse model, intraperitoneal DDN administration significantly reduced macrophage oxidative burden, ROS generation, and M1 polarization, ultimately mitigating collagen deposition and fibrotic progression. These findings establish DDN as a potent and targeted therapeutic platform for liver fibrosis treatment.

## Graphical abstract

This study developed a dynamic DNA nano‐antioxidant (DDN) for Galectin‐3 siRNA delivery, specifically targeting hepatic macrophages to suppress ROS‐driven fibrosis. DDN enhanced hepatic accumulation, restored NRF2 signaling, and attenuated oxidative stress, collagen deposition, and fibrotic progression in a CCl_4_‐induced cirrhosis model, demonstrating targeted therapeutic potential.

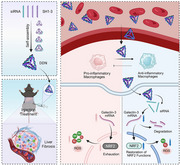

## 1 Introduction

Liver cirrhosis represents a major global health burden, accounting for millions of annual deaths worldwide.^[^ ^1^, ^2^ ^]^ Despite considerable advances in elucidating its pathogenic mechanisms and developing potential therapies, no effective treatment for liver cirrhosis has been successfully translated into clinical practice. Accumulating evidence indicates that persistent and recurrent inflammatory injuries play a pivotal role in driving fibrotic progression.^[^ ^3^, ^4^ ^]^ Notably, oxidative stress in hepatic macrophages triggers excessive release of pro‐inflammatory cytokines and reactive oxygen species (ROS), serving as a crucial initiating event in hepatic inflammation.^[^ ^5^, ^6^ ^]^ Thus, therapeutic strategies aimed at modulating macrophage M1 polarization and attenuating ROS production may offer a viable approach to mitigate liver cirrhosis.

Galectin‐3, a β‐galactoside‐binding lectin belonging to the galectin family, exhibits dynamic subcellular localization, shuttling between the cytoplasm and nucleus.^[^ ^7^, ^8^ ^]^ Previous studies demonstrate a strong positive correlation between Galectin‐3 expression levels in macrophages and the progression of liver fibrosis.^[^ ^9^, ^10^ ^]^ Preclinical studies using genetic knockout models or pharmacological inhibition of Galectin‐3 have shown significant attenuation of liver fibrosis and hepatic inflammation in murine models.^[^ ^11^, ^12^, ^13^ ^]^ Early‐phase clinical evaluation of the small‐molecule Galectin‐3 inhibitor Belapectin (GR‐MD‐02) yielded promising results, with significant improvements in FibroTest scores.^[^ ^14^ ^]^ However, subsequent Phase II clinical investigations revealed no significant therapeutic benefits of Belapectin in ameliorating either histological fibrosis or portal hypertension, likely attributable to its limited specificity.^[^ ^11^, ^15^, ^16^ ^]^ In this context, RNA interference‐mediated gene silencing serves as a superior therapeutic strategy, as small interfering RNA (siRNA) offers unparalleled target selectivity compared to conventional small‐molecule inhibitors, positioning Galectin‐3 targeting siRNA as a promising therapeutic modality for liver fibrosis.^[^ ^17^, ^18^ ^]^

Our previous engineered tetrahedral framework nucleic acid (tFNA), demonstrates exceptional capabilities for siRNA delivery, characterized by remarkable stability, high synthesis yield, and excellent biocompatibility.^[^ ^19^, ^20^ ^]^ More importantly, the intrinsic nucleic acid composition of tFNA confers an inherent capacity for efficient ROS clearance.^[^ ^21^, ^22^ ^]^ These distinctive properties render tFNA a particularly prospective therapeutic platform for combating liver cirrhosis. Our preliminary investigations revealed that intraperitoneal (i.p.) administration of tFNA resulted in its preferential hepatic accumulation and subsequent efficient uptake by macrophages.^[^ ^20^, ^23^ ^]^ This targeted delivery mechanism enables tFNA to function as a bifunctional therapeutic agent, capable of simultaneously facilitating target gene silencing through siRNA delivery and ameliorating oxidative stress via ROS scavenging in macrophages. To enhance structural stability, we proposed a novel strategy involving the site‐specific conjugation of Galectin‐3‐targeting siRNA onto the edges of tFNA, thereby constructing a dynamic DNA nano‐antioxidant (DDN).^[^ ^21^ ^]^ This rationally designed DDN exhibits dual functionality, enabling simultaneous Galectin‐3 silencing in macrophages and efficient ROS scavenging.

In this study, we employed single‐cell RNA sequencing (scRNA‐seq) to delineate the precise role of Galectin‐3 in fibrogenesis. We designed siRNA and successfully synthesized DDN to achieve the specific inhibition of Galectin‐3 in macrophages. Systematically elucidated the molecular mechanisms underlined DDN‐mediated suppression of macrophage activation, pro‐inflammatory signaling, and ROS production. Furthermore, therapeutic efficacy following i.p. administration of DDN was demonstrated by a carbon tetrachloride (CCl_4_)‐induced mouse model. Collectively, our findings not only validate DDN as a potential anti‐fibrotic therapy but also establish an innovative functional tetrahedral DNA‐based therapeutic paradigm for clinical translation in liver cirrhosis.

## 2 Results

### 2.1 Upregulation of Galectin‐3 in Liver Fibrosis

**Figure 1:**
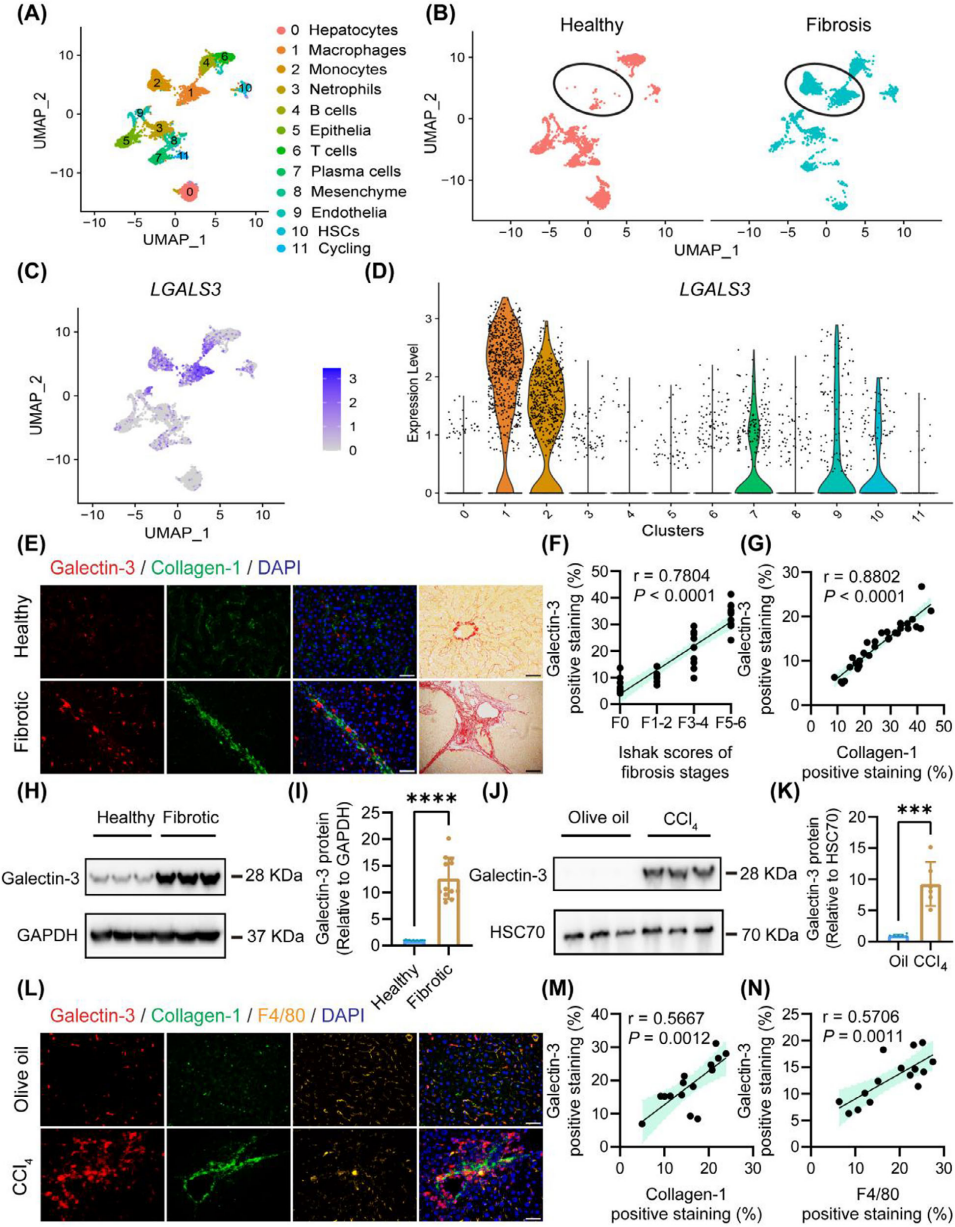
Galectin‐3 in macrophages promoted liver fibrosis. A) 12 clusters of 66135 cells from healthy (n = 5) and fibrotic (n = 5) livers; B) Clustering 66135 cells from healthy (n = 5) and fibrotic (n = 5) human livers, annotating injury condition; C) The expression of *LGALS3* in the 66135 cells; D) The expression of *LGALS3* in different clusters of cells; E) Representative immunofluorescence images of Galectin‐3, collagen‐1, and DAPI in both healthy and fibrotic livers. Representative Sirius red images in both healthy and fibrotic livers (right). Scale bar = 50 µm; F) Graphs showed the correlation between the Galectin‐3 positive staining and collagen‐1 positive staining in healthy (n = 11) and fibrotic (n = 16) human livers; G) Graphs showed the correlation between the Galectin‐3 positive staining and Ishak scores in healthy (n = 7) and fibrotic (n = 10) human livers; H) Representative western blotting images of Galectin‐3 expression in healthy (n = 3) and fibrotic (n = 3) human livers; I) Statistic analysis of (H). Data are presented as the mean ± SD (n = 12). ^***^ *P* < 0.001; J) Representative western blotting images and statistical analysis of Galectin‐3 expression in olive oil (n = 3) and CCl_4_ (n = 3) treated mouse livers; K) Statistical analysis of (J). Data are presented as the mean ± SD (n = 3). ^****^ *P* < 0.0001; L) Representative immunofluorescence images of Galectin‐3, collagen‐1, F4/80 and DAPI in both olive oil and CCl_4_ treated mouse livers. Scale bar = 50 µm; M) Graphs showed the correlation between the Galectin‐3 positive staining and collagen‐1 positive staining in olive oil (n = 7) and CCl_4_ (n = 8) treated mouse livers; N) Graphs showed the correlation between the Galectin‐3 positive staining and F4/80 positive staining in olive oil (n = 7) and CCl_4_ (n = 8) treated mouse livers.

To identify the key driver gene of liver fibrosis, we conducted a reanalysis scRNA‐seq data from healthy and fibrotic human liver (GSE136103). All cells were segregated into 12 transcriptionally distinct clusters (**Figure** **1**A). Comparative assessment revealed a significant expansion of monocyte clusters and macrophage clusters in fibrotic livers (Figure 1B). Differential gene expression analysis within the macrophage identified *LGALS3* (encoding Galectin‐3) as one of the most prominently upregulated genes in liver fibrosis (Figure S1, Supporting Information). Cellular mapping further demonstrated that *LGALS3* expression was highly enriched in monocyte clusters and macrophage clusters (Figure 1C,D), with significantly elevated transcript levels in fibrotic versus healthy livers.

To enhance these findings at the protein level, we performed immunofluorescence staining on the human liver samples. Galectin‐3 exhibited pronounced perifibrotic localization, predominantly encircling collagen‐1 deposited regions (Figure 1E). The expression of Galectin‐3 showed strong positive correlations with both collagen‐1 (r = 0.75, *P* < 0.0001, Figure 1F) and histological Ishak fibrosis scores (r = 0.85, *P* < 0.0001, Figure 1G). Western blot analysis confirmed that Galectin‐3 expression in fibrotic human livers was ≈13.25‐fold higher than that in healthy livers (Figure 1H,I). Parallel investigations in a mouse CCl_4_‐induced fibrosis model. Western blotting revealed an 8.78‐fold upregulation of Galectin‐3 in CCl_4_ treated livers versus olive oil treated livers (Figure 1J,K). Immunofluorescence co‐staining revealed Galectin‐3 overexpression specifically in F4/80^+^ macrophages infiltrating fibrotic areas (Figure 1L). Quantitative analyses demonstrated significant correlations between Galectin‐3 intensity and both collagen‐1 (r = 0.57, *P =* 0.0012, Figure 1M) and F4/80 (r = 0.57, *P =* 0.0011, Figure 1N).

To further elucidate the mechanistic role of Galectin‐3 in macrophage‐mediated inflammation, we established an in vitro inflammatory model using lipopolysaccharide (LPS) and interferon‐γ (IFN‐γ) stimulated RAW264.7 cell line (mouse leukemia cells of macrophages). RNA‐seq revealed significant upregulation of both *Lgals3* and *Nrf2* following LPS and IFN‐γ stimulation compared to controls (Figure S2A, Supporting Information). Quantitative validation by qRT‐PCR demonstrated a time‐dependent modulation of *Nrf2* expression, with maximal induction observed at 3 h post‐stimulation, followed by progressive attenuation with prolonged exposure (Figure S2B, Supporting Information). This temporal pattern suggested stimulus‐dependent exhaustion of *Nrf2* under chronic inflammatory conditions. Parallel analysis of *Lgals3* expression revealed peak transcript levels at 12 h post‐stimulation (Figure S2C, Supporting Information).

Based on the distinct cellular localization of Galectin‐3 (a cell surface receptor) and NRF2 (a nuclear transcription factor), we postulated that LPS and IFN‐γ induced Galectin‐3 mediates NRF2 exhaustion, thereby promoting oxidative stress in macrophages (Figure S2D, Supporting Information). Western blot analysis confirmed this hypothesis, showing NRF2 protein levels peaking at 6 h before declining significantly with extended stimulation (Figures S3A and S3B, Supporting Information). Corroborating these findings, immunofluorescence and flow cytometry (FCM) analysis demonstrated markedly elevated ROS release following 12 h of LPS and IFN‐γ treatment (Figures S3C–F, Supporting Information). To comprehensively investigate the antioxidant mechanisms, we systematically analyzed NRF2‐mediated transcriptional networks. Three *Nrf2* related gene databases (GEO and STRING) identified 56 downstream targets through Venn analysis (Figure S4A, Supporting Information). These genes revealed four principal antioxidant response elements that exhibited coordinated expression patterns: carbonyl reductase 3 (Cbr3), nicotinamide nucleotide transhydrogenase (Nnt), heme oxygenase‐1 (Hmox1), and NAD(P)H quinone dehydrogenase 1 (Nqo1) (Figure S4B, Supporting Information). In line with *Nrf2*, the expression of these antioxidant genes was significantly decreased following LPS and IFN‐γ stimulation of 3 h (Figure S5, Supporting Information).

Collectively, our investigation provides evidence for the pathogenic role of Galectin‐3 in liver fibrosis progression through NRF2 pathway dysregulation in macrophage‐mediated mechanisms. These results position Galectin‐3 inhibition as a potentially viable therapeutic approach for liver fibrosis.

### 2.2 Screening of Galectin‐3 Targeting siRNA and Characterization of DDN

**Figure 2:**
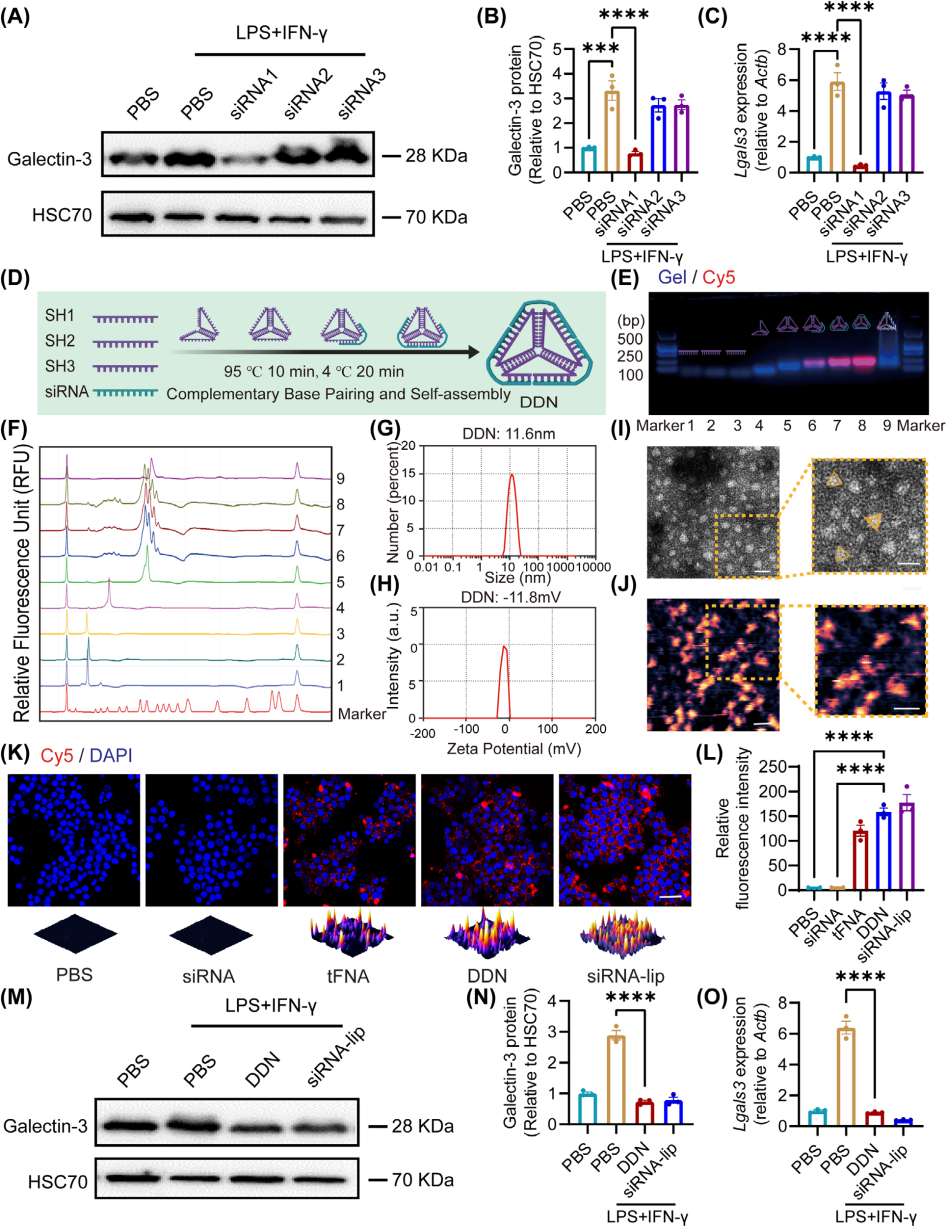
Preparation and characterization of DDN.A) RAW264.7 cells were transfected with PBS, siRNA1, siRNA2, or siRNA3, then treated with PBS or LPS + IFN‐γ. Representative western blotting images of Galectin‐3 expression in each group; B) Statistical analysis of (A). Data are presented as the mean ± SD (n = 3). ^***^ *P* < 0.001, ^****^ *P* < 0.0001; C) The expression of *Lgals3* in each group. Data are presented as the mean ± SD (n = 3). ^****^ *P* < 0.0001; D) Schematic illustration of the self‐assembly synthesis process of DDN; E) AGE showed the stepwise construction of the DDN. Lane 1: SH1; lane 2: SH2; lane 3: SH3; lane 4: SH1+ SH2; lane 5: SH1+ SH2+SH3; lane 6: SH1+ SH2+SH3+ siRNA; lane 7: SH1+ SH2+SH3+ 2 siRNA; lane 8: SH1+ SH2+SH3+ 3 siRNA (DDN); lane 9: tFNA. Red: Cy5 channel; blue: Gel Red channel; F) High‐performance capillary electrophoresis (HPCE) showed the stepwise construction of the DDN. The order of lanes 1–9 is the same as (E); G) The size of DDN; H) The ζ potential of DDN; I) The TEM images of DDN. Scale bar = 20 nm; J) The AFM images of DDN. Scale bar = 20 nm; K) The fluorescence images of RAW264.7 cells that were treated with PBS, Cy5‐siRNA, Cy5‐tFNA, Cy5‐DDN, or Cy5‐siRNA‐lip at 6 h. Scale bar = 25 µm; L) Statistic analysis of (K). Data are presented as the mean ± SD (n = 3). ^****^ *P* < 0.0001; M) RAW264.7 cells were transfected with PBS, DDN, or siRNA‐lip, then treated with PBS or LPS + IFN‐γ. Representative western blotting images of Galectin‐3 expression in each group; N) Statistic analysis of (M). Data are presented as the mean ± SD (n = 3). ^****^ *P* < 0.0001; O) The expression of *Lgals3* of in each group. Data are presented as the mean ± SD (n = 3). ****P < 0.0001.

In pursuit of Galectin‐3 inhibition to ameliorate macrophage‐mediated inflammation and fibrotic progression, we systematically designed and evaluated three distinct siRNAs targeting *Lgals3*. Quantitative Western blot analysis revealed siRNA1 as the most efficacious construct, demonstrating a significant 3.21‐fold reduction in Galectin‐3 expression (*P* < 0.0001) compared to controls, while siRNA2 and siRNA3 showed negligible effects (**Figure** **2**A,B). This suppression was corroborated at the transcriptional level, with siRNA1 achieving a remarkable 6.18‐fold decrease in *Lgals3* expression (*P* < 0.001, Figure 2C).

To establish an efficient nucleic acid delivery system, we constructed tFNAs and confirmed the enhanced cellular internalization. Immunofluorescence demonstrated a statistically significant improvement in cellular uptake efficiency for tFNAs relative to single strands DNAs (ssDNA) (*P* < 0.0001, Figure S6, Supporting Information). Building upon this foundation, we developed DDN specifically designed for siRNA delivery (Figure 2D). Stepwise assembly was rigorously verified through agarose gel electrophoresis (AGE), confirming the successful incorporation of SH1, SH2, and SH3 structural components with the siRNA payload (Figure 2E,F). Nanostructural characterization using transmission electron microscopy (TEM) and atomic force microscopy (AFM) revealed DDN with a mean hydrodynamic diameter of 11.6 nm (Figure 2G,I). Surface potential analysis by dynamic light scattering recorded a ζ‐potential of ‐11.8 mV (Figure 2H,J). Notably, immunofluorescence demonstrated DDN's superior performance, exhibiting a 1.76‐fold enhancement in cellular uptake efficiency relative to tFNAs (*P* < 0.0001, Figure 2K,L). DDN‐mediated siRNA delivery achieved a significant 2.94‐fold reduction in Galectin‐3 expression (*P* < 0.001) and an impressive 11.75‐fold suppression at the mRNA level (*P* < 0.001), as determined by Western blot and RT‐qPCR, respectively (Figure 2M–O).

### 2.3 Reduction of Oxidative Stress by DDN via Restoration the Exhaustion of NRF2 in Macrophages

**Figure 3:**
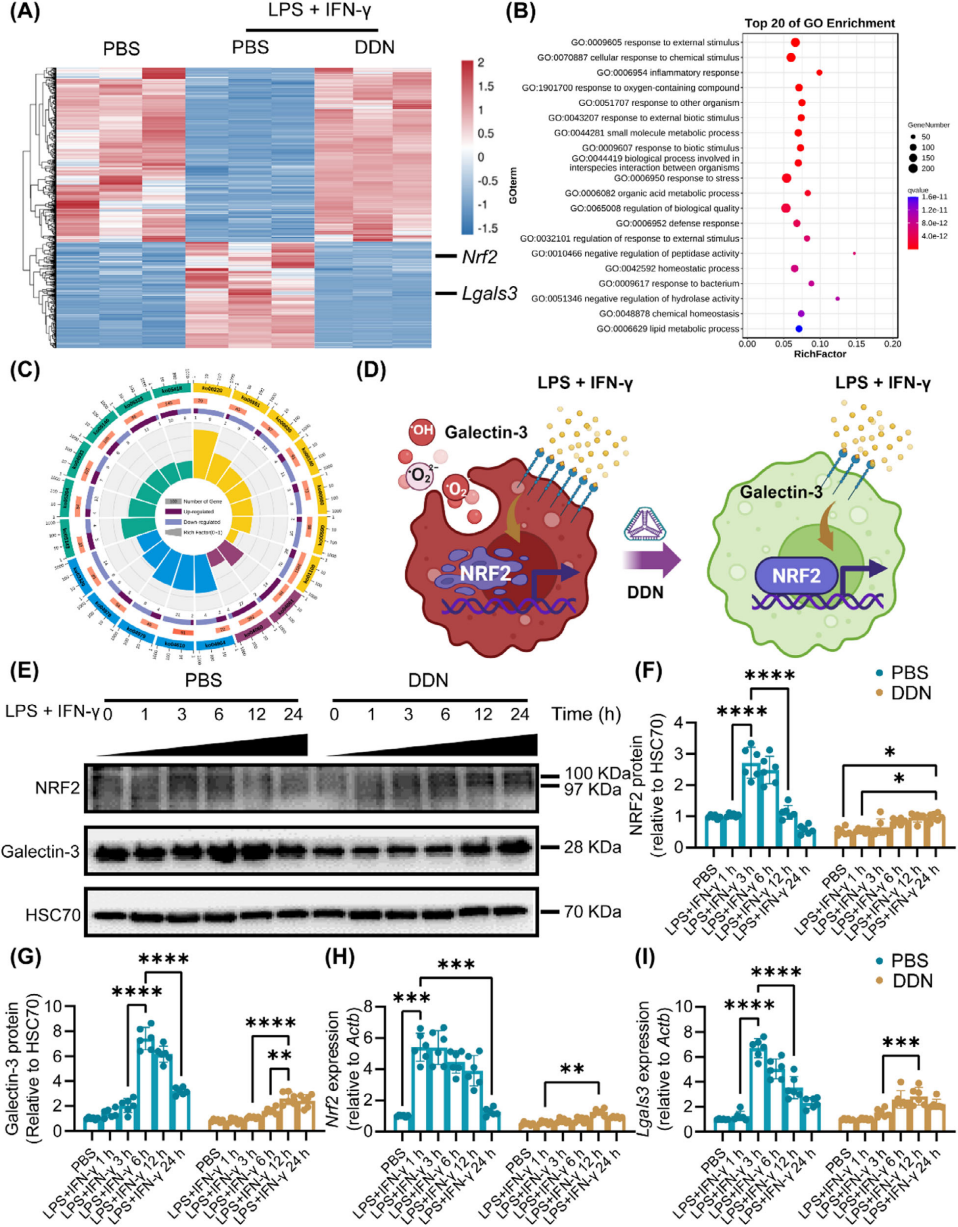
DDN restrained the exhaustion of NRF2 via silencing Galectin‐3 expression. A) RAW264.7 cells were treated with PBS or DDN, then stimulated with LPS + IFN‐γ. The RAW264.7 cells were collected and performed to RNA‐seq. Heatmaps of differentially expressed genes among the groups; B) Bubble diagram showed the top 20 significantly enriched GO terms; C) Petal diagram showed the top 20 significantly enriched GO terms; D) Schematic illustration of DDN‐mediated reduction in LPS and IFN‐γ induced macrophage oxidative stress and ROS release through Galectin‐3 suppression and subsequent prevention of NRF2 depletion; E) RAW264.7 cells were treated with PBS or DDN, then stimulated with LPS + IFN‐γ by different time points. Representative western blotting images of Galectin‐3 and NRF2 expression in each group; F,G) Statistic analysis of (K). Data are presented as the mean ± SD (n = 6). ^*^ *P* < 0.05, ^**^ *P* < 0.01, ^****^ *P* < 0.0001; H,I) The expression of *Lgals3* and *Nrf2* in each group. Data are presented as the mean ± SD (n = 6). ^**^ *P* < 0.01, ^***^ *P* < 0.001, ^****^ *P* < 0.0001.

Comparative transcriptome analysis was conducted across three experimental groups, including PBS‐treated controls, LPS and IFN‐γ stimulated macrophages, and DDN‐treated macrophages following LPS and IFN‐γ stimulation. Differential gene analysis revealed significant downregulation of both *Nrf2* and *Lgals3* in the DDN‐treated group compared to the LPS and IFN‐γ stimulated group (**Figure** **3**A). Gene Ontology (GO) enrichment analysis demonstrated a predominant association of differentially expressed genes with oxidative stress response and inflammatory pathways (Figure 3B,C). These results supported the hypothesis that DDN attenuated ROS production by inhibiting Galectin‐3‐mediated NRF2 depletion (Figure 3D). Time‐course quantification of NRF2 protein expression revealed distinct kinetic profiles. LPS and IFN‐γ stimulation induced NRF2 elevation with peaking at 3 h, followed by progressive decrease. However, DDN treatment suppressed NRF2 expression (Figure 3E–G). Consistently, qRT‐PCR confirmed this preservation effect of DDN treatment (Figure 3H,I).

**Figure 4:**
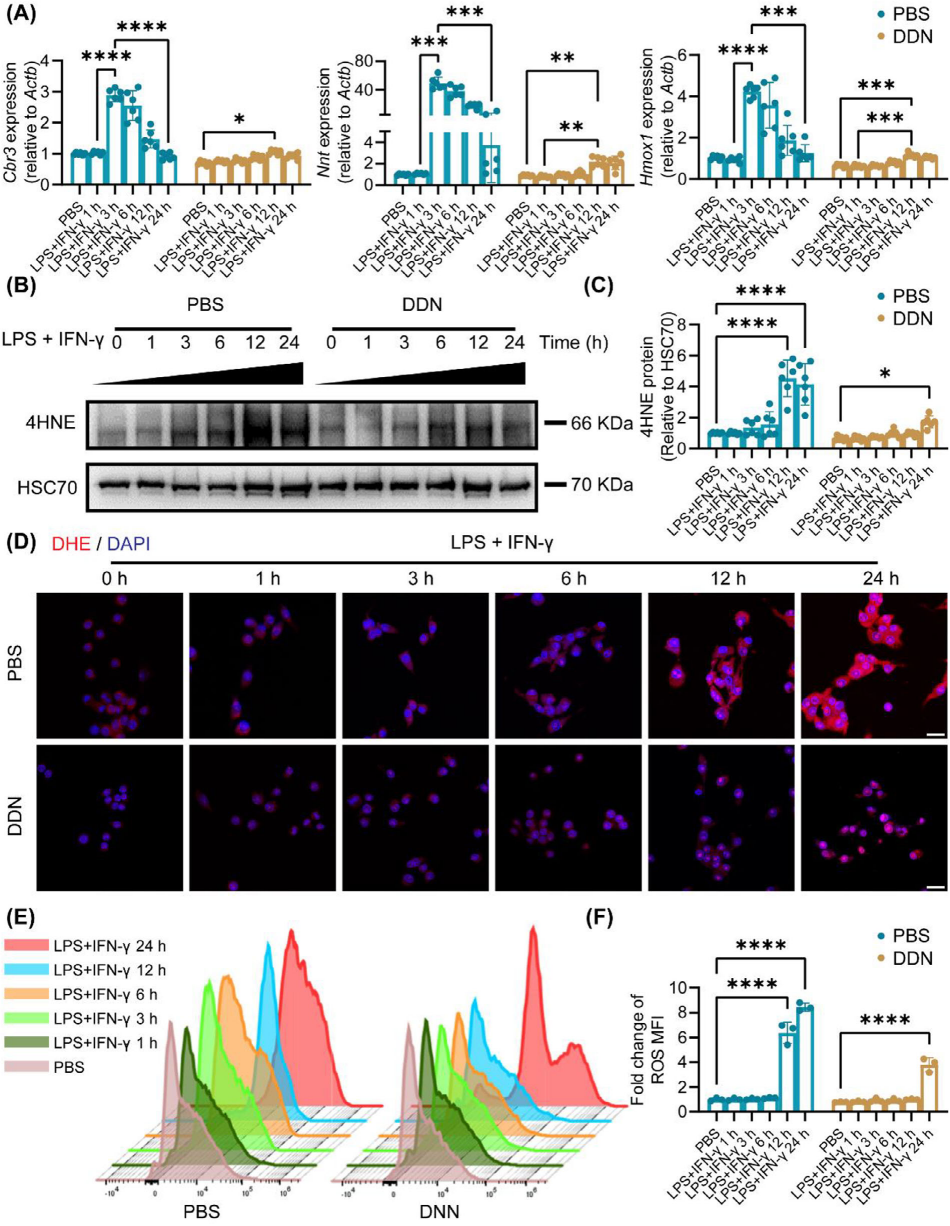
DDN alleviated the oxidative stress and ROS release in macrophages. A) RAW264.7 cells were treated with PBS or DDN, then stimulated with LPS + IFN‐γ by different time points. The expression of *Cbr3*, *Nnt2*, and *Hmox1* in each group; B) Representative western blotting images of 4HNE expression in each group; C) Statistical analysis of (B), Data are presented as the mean ± SD (n = 6). ^*^ *P* < 0.05, ^****^ *P* < 0.0001; D) Representative immunofluorescence images of DHE and DAPI in each group, scale bar = 25 µm; E) Representative flow cytometry images of ROS in each group; F) Statistic analysis of (E), Data are presented as the mean ± SD (n = 6). ^****^ *P* < 0.0001.

To systematically evaluate the antioxidant capacity of DDN, we examined the expression of the oxidant response‐related genes, including *Cbr3*, *Nnt*, *Hmox1*, and *Nqo1*. Time‐course analysis revealed that while LPS and IFN‐γ stimulation induced progressive increase (peaking at 3 h), DDN treatment decreased the sustained expression of these antioxidant genes (**Figure** **4**A; Figure S7, Supporting Information). In order to demonstrate the level of oxidative stress, we examined the expression of 4‐hydroxynonenal (4HNE), a lipid peroxidation biomarker.^[^ ^24^ ^]^ Quantitative analysis revealed that LPS and IFN‐γ stimulation induced a significant increase in 4HNE levels by 12 h, indicative of pronounced lipid peroxidation. Notably, DDN treatment delayed this oxidative stress response, with 4HNE elevation reaching significance only at 24 h (Figure 4B,C). Complementary flow cytometric and immunofluorescence analyses demonstrated that DDN significantly attenuated ROS release compared to the LPS and IFN‐γ stimulated group (Figure 4D–F). These findings provide compelling evidence that DDN exerts its antioxidant effects through effective suppression of Galectin‐3 expression and preservation of NRF2 levels.

### 2.4 Accumulation of DDN in Liver Macrophages In Vivo

**Figure 5:**
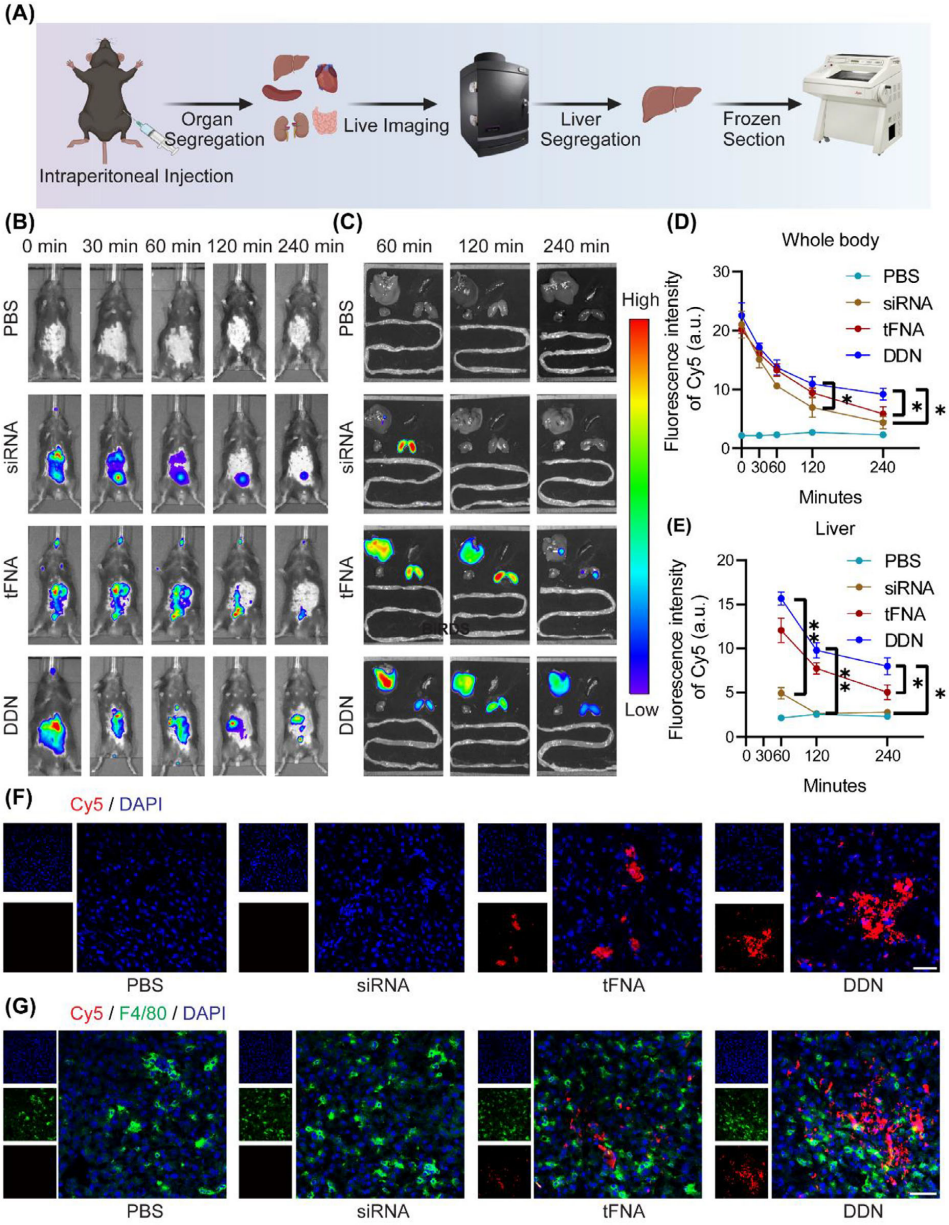
Liver‐specific delivery of DDN. A) Scheme illustration of IVIS and immunofluorescence imaging after i.p. administration of DDN; B) In vivo fluorescence images of the whole body were taken at different time points after i.p. administration of PBS, Cy5‐siRNA, Cy5‐tFNA, and Cy5‐DDN. C) In vivo fluorescence images of the isolated organs (liver, spleen, heart, kidney, and intestine) were taken at different time points after i.p. administration of PBS, Cy5‐siRNA, Cy5‐tFNA, and Cy5‐DDN. D) Quantitative analysis of Cy5 fluorescence intensities of the whole body in each group. Data are presented as the mean ± SD (n = 6). ^*^ *P* < 0.05; E) Quantitative analysis of Cy5 fluorescence intensities of liver in each group. Data are presented as the mean ± SD (n = 6). ^*^ *P* < 0.05, ^**^ *P* < 0.01; F) Representative immunofluorescence images of Cy5 and DAPI in liver, scale bar = 50 µm; G) Representative immunofluorescence images of Cy5, F4/80, and DAPI in liver, scale bar = 50 µm.

Comprehensive evaluation of tFNA biodistribution following i.p. injection in mice revealed significant pharmacokinetic advantages over ssDNA (**Figure** **5**A). IVIS imaging demonstrated markedly prolonged systemic retention of tFNAs (Figure S8A, Supporting Information). Quantitative organ imaging identified preferential hepatic accumulation of tFNAs, with greater fluorescence intensity in liver tissue relative to ssDNA (Figure S8B, Supporting Information). Immunofluorescence analysis of liver cryosections further confirmed the enhanced stability profile of tFNAs (Figure S8C, Supporting Information). These results collectively established the structural superiority of tetrahedral architectures for targeted hepatic delivery.

Subsequent characterization of DDN demonstrated further optimized pharmacokinetic properties. Whole‐body IVIS imaging revealed greater fluorescence intensity for DDN compared to either siRNA or tFNA at 240 min after i.p. injection (*P* < 0.05, Figure 5B,D), with organ imaging showing 1.57‐ and 2.11‐fold greater fluorescence intensity at 240 min post‐injection compared to siRNA and tFNA (*P* < 0.05, Figure 5C,E). Notably, DDN maintained detectable hepatic signals beyond 240 min, compared to 120 min for siRNA and tFNA groups (Figure 5C,E). High‐resolution immunofluorescence imaging of liver sections confirmed both nanostructure stability and specific colocalization with F4/80^+^ Kupffer cells (Figure 5F,G), demonstrating the successful achievement of macrophage‐targeted delivery.

These findings proved the inherent hepatic tropism of DDN and their specific targeting capability to liver macrophages. The quantitative biodistribution data, acquired through various imaging modalities, provided robust validation of DDN's delivery efficiency and target specificity, supporting their therapeutic potential for liver‐directed applications.

### 2.5 Alleviation of Fibrosis by DDN in CCl_4_‐Induced Liver Fibrosis Mouse

**Figure 6:**
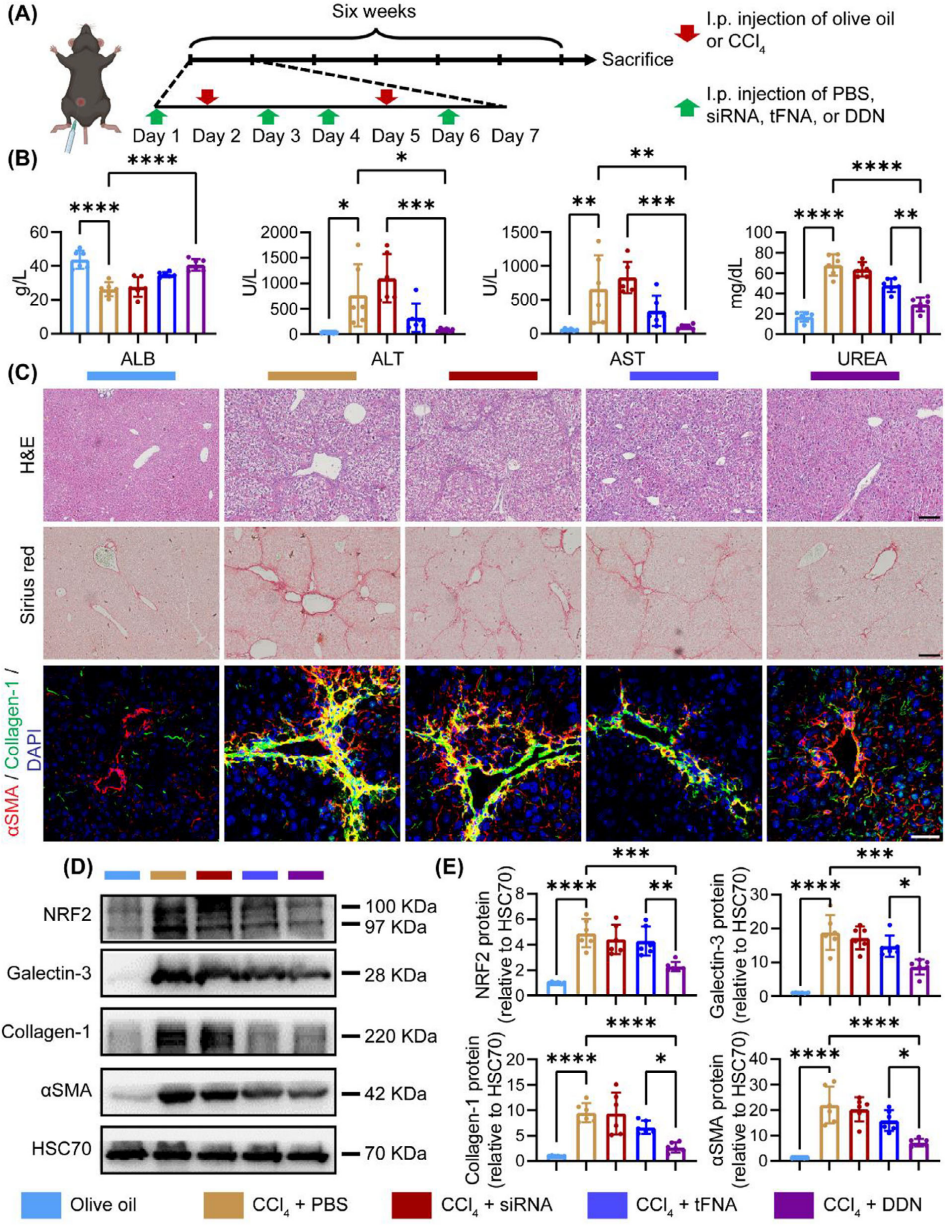
Reduction of liver fibrosis by DDN.A) Schematic illustration of the treatment protocol in the chronic liver fibrosis mouse model; B) Effects of different treatments (Olive oil, CCl_4_ + PBS, CCl_4_ + siRNA, CCl_4_ + tFNA, CCl_4_ + DDN) on liver function indicators, including ALB, ALT, AST, and UREA levels; C) Representative H&E staining image, Sirius red staining images and immunofluorescence images of αSMA, collagen‐1 and DAPI of liver in each group; black scale bar = 100 µm, white scale bar = 50 µm; D) Representative western blotting images of NRF2, Galectin‐3, collagen‐1, and αSMA expression in each group; E) Statistic analysis of (D), Data are presented as the mean ± SD (n = 6). ^**^ *P* < 0.01, ^***^ *P* < 0.001, ^****^ *P* < 0.0001.

To investigate the therapeutic potential of DDN in liver fibrosis, we employed a CCl_4_‐induced murine fibrosis model. Mice were co‐administered CCl_4_ alongside siRNA, tFNA, or DDN (**Figure** **6**A). Biochemical profiling demonstrated that DDN treatment significantly ameliorated liver injury, as evidenced by reduced serum levels of alanine aminotransferase (ALT) (*P* < 0.05), aspartate aminotransferase (AST) (*P* < 0.01), and blood urea nitrogen (UREA) (*P* < 0.0001) and elevated albumin (ALB) (*P* < 0.0001) compared to the CCl_4_ treated group (Figure 6B). Histopathological assessment via Hematoxylin and Eosin (H&E) and Sirius red staining confirmed the anti‐fibrotic effects of DDN, revealing a marked reduction in collagen deposition and pseudolobule formation (Figure 6C). Immunofluorescence analysis corroborated these findings, showing diminished expression of αSMA and collagen‐1 in DDN‐treated mice (Figure 6C). Western blot of liver tissue lysates revealed a significant downregulation of Galectin‐3 in the DDN group compared to the CCl_4_ group. Furthermore, fibrosis‐associated proteins, including collagen‐1 and αSMA, were substantially suppressed following DDN treatment (*P* < 0.001) (Figure 6D,E).

**Figure 7:**
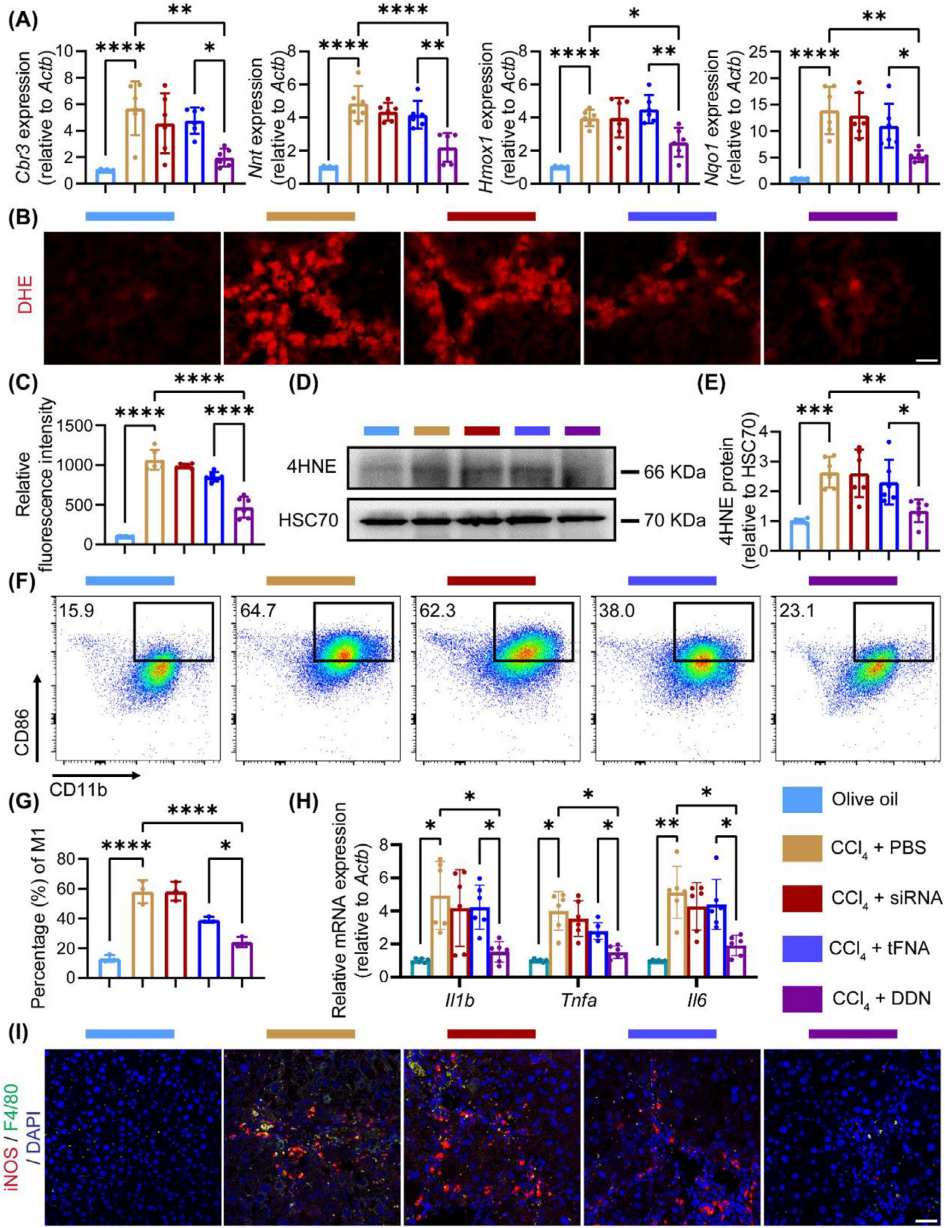
Inhibition of inflammation and oxidative stress by DDN.A) Effects of different treatments (Olive oil, CCl_4_ + PBS, CCl_4_ + siRNA, CCl_4_ + tFNA, CCl_4_ + DDN) on antioxidant genes, including *Cbr3*, *Nnt2*, *Hmox1*, and *Nqo1*; B) Representative immunofluorescence images of DHE in each group, scale bar = 50 µm; C) Quantitative analysis of DHE fluorescence intensities of liver in each group. Data are presented as the mean ± SD (n = 6). ^***^ *P* < 0.001, ^****^ *P* < 0.0001; D) Representative western blotting images of 4HNE in each group; E) Statistic analysis of (D), Data are presented as the mean ± SD (n = 6). ^**^ *P* < 0.01, ^***^ *P* < 0.001; F) Representative flow cytometry images of M1 macrophage subpopulation in different groups. G) Statistic analysis of (F). Data are presented as the mean ± SD (n = 3). ^****^ *P* < 0.0001; H) Expression of sentinel proinflammatory genes including *Il1b*, *Tnfa*, and *Il6* in each group. Data are presented as the mean ± SD (n = 6). ^*^ *P* < 0.05, ^**^ *P* < 0.01; I) Representative immunofluorescence images of iNOS, F4/80, and DAPI in each group, scale bar = 25 µm.

Given the pivotal role of oxidative stress and inflammation in fibrosis progression, we next evaluated the impact of DDN on these pathways in vivo. Western blot analysis indicated a pronounced decrease in NRF2 expression in DDN‐treated mice (*P* < 0.01), accompanied by reduced levels of downstream antioxidative mediators (*Cbr3*, *Nnt*, *Hmox1*, and *Nqo1*) (*P* < 0.05, Figure 6D,E and **Figure** **7**A). Hepatic oxidative stress was further assessed via Dihydroethidium (DHE) immunofluorescence and 4HNE immunoblotting, both of which exhibited significantly lower signals in the DDN group (*P* < 0.01, Figure 7B–E). Furthermore, we isolated hepatic nonparenchymal cells (NPCs) and quantified macrophage polarization by flow cytometry to delineate the inflammatory response. Strikingly, DDN administration led to a substantial reduction in M1‐polarized macrophages compared to the CCl_4_ group (*P* < 0.0001, Figure 7F,G). Consistent with this observation, qPCR analysis revealed the downregulation of pro‐inflammatory cytokines (*Il1b*, *Tnfa*, and *Il6*) in DDN‐treated mice (Figure 7H). Immunofluorescence staining further validated these results, demonstrating attenuated expression of iNOS, IL‐1β, TNF‐α, and IL‐6 in hepatic macrophages (Figure 7I; Figure S9, Supporting Information).

To assess potential off‐target effects, we conducted histopathological examinations of major organs, including the lung, heart, stomach, spleen, kidney, and intestine among the groups. No discernible morphological alterations were observed in any of these tissues (Figure S10, Supporting Information), underscoring the favorable biocompatibility and minimal systemic toxicity of DDN.

## 3 Discussion

In this study, the scRNA‐seq analysis established Galectin‐3 as an essential regulator in the pathogenesis of liver cirrhosis, with a predominant role in macrophage‐driven fibrogenesis. A novel mechanistic pathway wherein Galectin‐3 orchestrates oxidative stress in macrophages through NRF2 exhaustion, consequently triggering excessive ROS generation. Through systematic screening and design, we developed DDN, an innovative siRNA‐based therapeutic platform specifically targeting Galectin‐3. The therapeutic efficacy of DDN was demonstrated through its ability to downregulate Galectin‐3 expression, thereby preserving NRF2 levels and ameliorating oxidative stress in macrophages. Notably, our in vivo studies proved that DDN achieved selective targeting of hepatic macrophages and conferred significant protection against liver fibrosis via Galectin‐3 suppression.

Liver cirrhosis represents a major determinant of morbidity and mortality worldwide, while simultaneously posing an enormous economic burden on healthcare systems.^[^ ^1^, ^25^ ^]^ While Galectin‐3 has been widely recognized as a promising molecular target for antifibrotic therapy, the clinical performance of conventional inhibitors has been disappointing.^[^ ^26^, ^27^ ^]^ Notably, Belapectin, a non‐selective Galectin‐3 inhibitor, failed to meet its primary endpoint in a Phase II clinical trial involving NASH patients, underscoring the inherent limitations of pan‐Galectin inhibition strategies.^[^ ^15^ ^]^ Our siRNA‐based approach represented paradigm improvements, offering specificity in Galectin‐3 suppression while circumventing the off‐target effects associated with small‐molecule inhibitors.^[^ ^28^, ^29^ ^]^ Furthermore, the transient nature of siRNA‐mediated gene silencing provides an additional safety advantage by minimizing the risk of chronic toxicity.^[^ ^30^ ^]^

Notwithstanding these advantages, the therapeutic application of siRNA remains constrained by substantial delivery challenges, particularly its rapid degradation in biological systems.^[^ ^31^ ^]^ The selection of an optimal delivery platform is therefore paramount to ensure both the stability and therapeutic efficacy of siRNA. In comparison, the current nanomaterials offer diverse delivery options, including lipid‐based systems, metal‐organic frameworks, and noble metal nanoparticles. However, these platforms may be suboptimal for hepatic applications. Specifically, the compromised metabolic capacity characteristic of fibrotic livers renders them particularly vulnerable to the potential hepatotoxicity of lipidic or metallic nanocarriers.^[^ ^21^, ^32^ ^]^ In contrast, tetrahedral DNA nanomaterials present distinct advantages, including exceptional biocompatibility, favorable biodegradation kinetics, and negligible hepatic toxicity accumulation.^[^ ^33^, ^34^ ^]^ Moreover, their inherent antioxidant properties provide an additional therapeutic dimension, making them ideally suited for addressing the oxidative stress component of liver fibrosis pathogenesis.^[^ ^21^, ^35^ ^]^

Tetrahedral DNA nanostructures have emerged as an excellent platform for siRNA delivery, offering unparalleled advantages, including exceptional biocompatibility, enhanced tissue penetrability, structural modularity, and minimal cytotoxicity.^[^ ^36^ ^]^ Our development of DDN, a second‐generation DNA nanostructures system, represents a superior advancement over the conventional tFNA. First, the strategic incorporation of siRNA conjugation along three structural edges enables complete base‐pair complementarity for all nucleotides, resulting in substantially improved stability compared to conventional vertex‐anchored configurations.^[^ ^19^ ^]^ Second, the unique architectural design incorporates dual protective elements (ssDNA and siRNA) at each vertex, conferring remarkable resistance to nuclease degradation.^[^ ^20^ ^]^ Third, the simplified structural topology permits more efficient large‐scale production with a great reduction in manufacturing costs while maintaining >95% assembly yield.^[^ ^19^ ^]^

The pronounced hepatic accumulation of DDN, as demonstrated by our IVIS imaging results, can be attributed to both passive and active targeting mechanisms. First, passive targeting plays a crucial role due to the liver's unique physiological characteristics. The liver sinusoids possess a discontinuous, fenestrated endothelium that permits efficient extravasation and retention of nanoparticles, particularly those within an optimal size range.^[^ ^37^ ^]^ Additionally, the liver receives a substantial portion of cardiac output, ensuring prolonged exposure to systemically administered nanoparticles.^[^ ^38^ ^]^ Second, active targeting further enhances DDN accumulation, as the liver serves as a major site for nucleic acid metabolism.^[^ ^39^ ^]^ Endogenous processes involved in oligonucleotide clearance and degradation may facilitate the selective uptake of DDN by hepatocytes and Kupffer cells. This dual‐targeting mechanism—passive accumulation due to hepatic physiology and active engagement with nucleic acid metabolic pathways—collectively contributes to the preferential liver distribution of DDN, thereby supporting its therapeutic potential for liver‐associated diseases.

The pathophysiological significance of oxidative stress in hepatic fibrogenesis has been well‐established, with compelling evidence demonstrating its role in promoting hepatic stellate cell activation and extracellular matrix deposition.^[^ ^40^, ^41^ ^]^ While macrophages have been identified as the predominant source of ROS release in fibrotic livers, current therapeutic strategies have failed to effectively target this pathogenic mechanism.^[^ ^42^, ^43^ ^]^ Our study provided insights into the Galectin‐3/NRF2 axis, revealing that macrophage‐derived Galectin‐3 drives oxidative stress through exhaustion of NRF2, the principal transcriptional regulator of antioxidant response elements. Most notably, DDN‐mediated Galectin‐3 silencing not only restored NRF2 protein levels but also significantly attenuated ROS production both in vitro and in vivo. These findings represented a promising strategy in fibrosis therapeutics, establishing DDN as a powerful tool for precise modulation of macrophage activation and oxidative stress.

## 4 Conclusion

**Figure 8:**
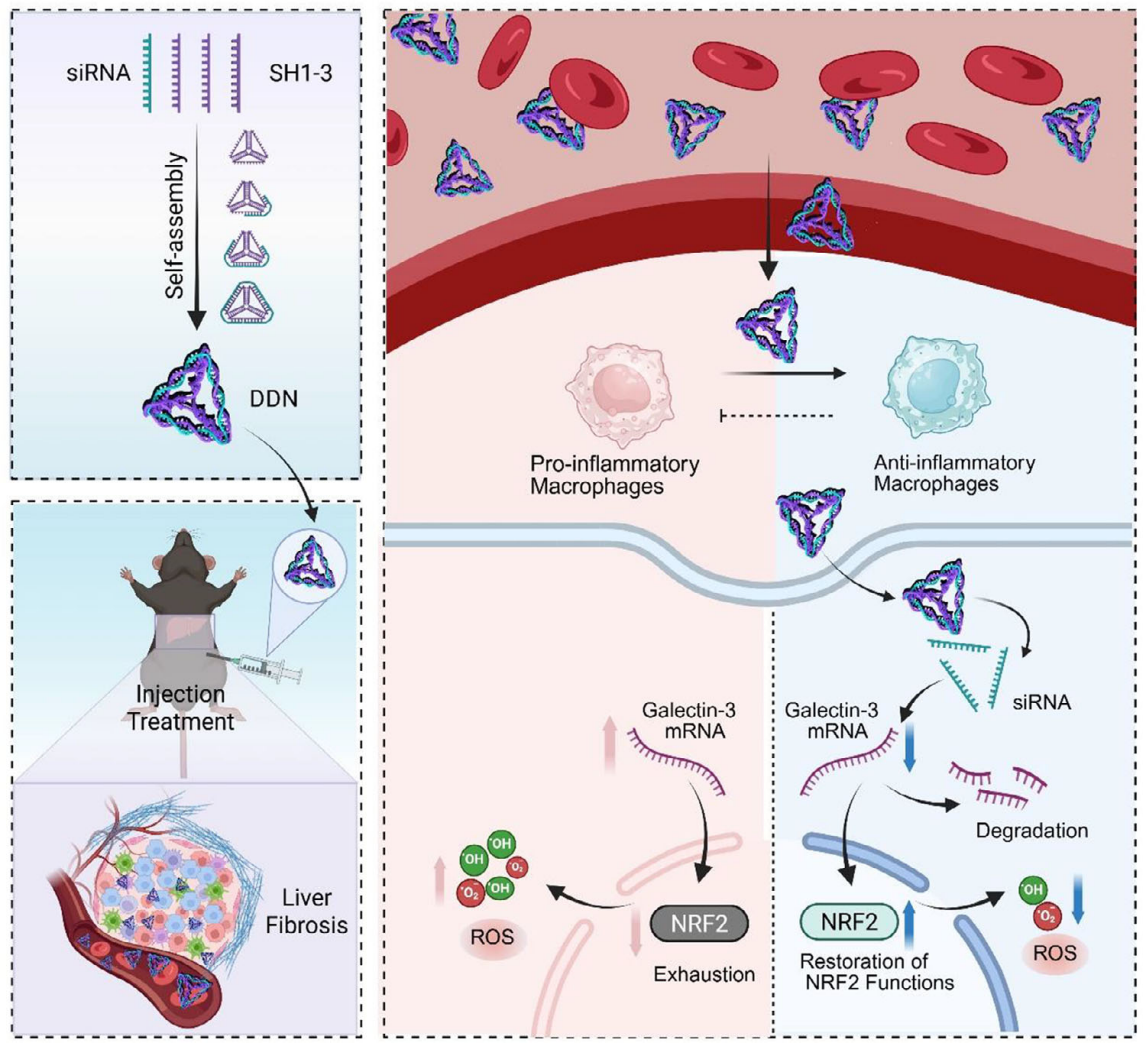
Proposed mechanisms of DDN. SH1, SH2, SH3, and siRNA undergo self‐assembly to form DDN, which are delivered via i.p. injection into the mesenteric venous system. Following entry into systemic circulation, DDN are preferentially transported via the portal venous system to the liver, where they are efficiently internalized by hepatic macrophages. Intracellular release of siRNA payload from DDN effectively downregulates expression of Galectin‐3, thereby preventing NRF2 depletion and restoring cellular antioxidant defense mechanisms. This process significantly reduces ROS generation and release. The consequent polarization of macrophages toward an anti‐inflammatory phenotype contributes to the amelioration of liver fibrosis.

scRNA‐seq analysis and functional validation study establish Galectin‐3 as an essential mediator in hepatic fibrogenesis. Through specimens of both patients and murine models, we demonstrated that Galectin‐3 expression in macrophages drives fibrotic progression. Based on the therapeutic potential of Galectin‐3, we designed and developed DDN, a novel siRNA‐based tetrahedral nanostructure, specifically silencing Galectin‐3 in macrophages. Mechanistic investigations combining bioinformatics approaches revealed that DDN exerts the anti‐fibrotic effects through the Galectin‐3/NRF2 axis, effectively mitigating oxidative stress ROS release in macrophages. Importantly, our in vivo therapeutic experiments demonstrated both the targeted delivery of DDN to liver macrophages and the efficacy for attenuating liver fibrosis in CCl_4_induced mouse model (**Figure** **8**). These findings not only advance our understanding of liver fibrosis pathogenesis but also present a promising targeted therapeutic strategy for clinical translation.

## 5 Experimental Section

### Preparation of tFNA and DDN

According to previous studies, tFNA was self‐assembled from four ssDNA with specific sequences (Table S1, Supporting Information) (Sangon, Shanghai, China).^[^ ^23^ ^]^ DDN was self‐assembled by siRNA and SH1‐SH3 (Tables S2 and S3, Supporting Information) (Sangon, Shanghai, China).^[^ ^19^ ^]^ The tFNA and DDN were synthesized by adding equimolar amounts of single ssDNA to the TM buffer (main components: 10 × 10^−3^ M Tris‐HCl and 50 × 10^−3^ M MgCl_2_) after two procedures of heating (95 °C, 10 min) and cooling (4 °C, 20 min).

### Characterization of tFNA and DDN

tFNA and DDN were validated via 2% AGE and capillary electrophoresis (Qsep100 Bioptic). The 1ˣTAE was added and performed at 120 V for 30 min and then exposed with a Bio‐Rad machine (Bio‐Rad, Hercules, USA). Dynamic light scattering (DLS, Nano ZS, Malvern, England) was used to measure the sizes and zeta potentials of the tFNAs and DDN. AFM (Cypher VRS, Oxford Instruments, United Kingdom) and TEM (Libra200, Zeiss, Oberkochen, Germany) were used to observe the morphology of the tFNAs and DDN.

### Induction of Liver Fibrosis Mouse Model

Eight‐week‐old C57BL/6 mice were purchased from Dossy (Chengdu, China) and housed under standard laboratory conditions. After delivery, mice were acclimatized for 2 weeks before experimental procedures to minimize stress‐induced variability. CCl_4_ (Sigma–Aldrich, USA) was diluted in olive oil (Macklin, China) at a 1:3 (v/v) ratio to prepare the working solution. The mixture was vortexed thoroughly and stored in light‐protected vials at 4 °C within 24 h.

For i.p. injections, mice were gently restrained by securing the head and tail to maintain alignment of the cervical, thoracic, and abdominal regions. The CCl_4_ was injected with 1 µL g^−1^ body weight into the lower right abdomen. Control mice received an equivalent volume of olive oil. CCl_4_ injections were performed twice weekly for 6 weeks to induce chronic liver fibrosis. Meanwhile, mice were administered either phosphate‐buffered saline (PBS, Gibco, USA), siRNA (1 mm), tFNA (1 mm), or DDN (1 mm) via i.p. injection in the lower left abdominal quadrant. Treatments were administered one day before and after each CCl_4_ injection (four times per week) for the entire 6‐week experimental period. Animal experiments were approved by the Ethics Committee of Sichuan University (20 241 009 006).

### Collection of Blood Samples of Mouse and Biochemical Testing

Blood samples were obtained through immediate enucleation under deep anesthesia. Whole blood was transferred into sterile 1.5 mL microcentrifuge tubes (Eppendorf, Germany) and allowed to coagulate at room temperature for 30 min. Clotted samples were centrifuged at 12000 × g for 10 min at 4 °C (Thermofisher, USA) to separate serum components. For biochemical analysis, 50 µL of serum was diluted with 150 µL of ice‐cold PBS to achieve a final 1:4 dilution. Quantification of serum biomarkers, including ALB, ALT, AST, and UREA, was performed using a fully automated clinical chemistry analyzer (Roche, USA) with manufacturer‐calibrated reagents.

### Collection of Mouse Organs

Following dissection, all harvested organs (liver, lung, heart, stomach, spleen, kidney, and intestine) were immediately fixed in freshly prepared 4% paraformaldehyde (PFA; Sigma‐Aldrich, USA) at room temperature for 2 days to ensure complete tissue preservation. To maintain optimal fixation quality, the PFA solution was replaced with a fresh fixative on day 1 of the fixation period. Fixed tissues were progressively dehydrated through a graded ethanol series and cleared using xylene. Cleared tissues were infiltrated with molten Paraplast Plus embedding medium. Paraffin‐embedded tissues were sectioned at 6 µm thickness using a rotary microtome (Leica, Germany). Serial sections were floated on a temperature‐controlled water bath to ensure complete expansion. Sections were processed through dewaxing and rehydration to prepare for the following histological staining.

### Histopathological Analysis of Mouse Organs

Tissue sections were stained using a standard H&E protocol according to the manufacturer's protocol (Beyotime, China). Briefly, sections were immersed in hematoxylin for nuclear staining, followed by differentiation in hydrochloric acid alcohol. Subsequently, bluing was performed in running tap water. Cytoplasmic staining was achieved using eosin. After dehydration through a graded ethanol series, the sections were cleared in xylene and mounted with neutral resin. Histological evaluation was conducted under a fluorescence microscope, and a minimum of three randomly selected fields per section were imaged for quantitative analysis. The Ishak scoring system, as previously described, was applied to assess the degree of liver fibrosis.^[^ ^44^, ^45^ ^]^

For collagen fiber detection, tissue sections were stained with 0.1% Sirius red dye (Beyotime, China) for 15 min. After thorough rinsing in running water, the sections were dehydrated in graded ethanol, cleared in xylene, and mounted with neutral resin. Collagen deposition was visualized and quantified under a fluorescence microscope, with at least three independent fields per section analyzed.

### Collection of Human Liver Tissue

The research procedure was performed in accordance with both the Declaration of Helsinki and Istanbul. The information of samples were listed in Table S4 (Supporting Information). The study was approved by the Ethical Committee of West China Hospital and registered in the Chinese Clinical Trial Registry. All of the patients provided written informed consent.

### Bioinformatic Analysis of the GEO Dataset

We utilized the Seurat software package (version 4.2.2) to analyze scRNA‐seq data from a published GEO dataset (GSE136103).^[^ ^46^ ^]^ This dataset comprises single‐cell transcriptome profiles obtained from five healthy and five cirrhotic livers. All the resident liver cells within the dataset were clustered into 11 distinct clusters via unsupervised learning algorithms implemented in Seurat. To identify genes differentially expressed across the identified clusters, we employed the FindMarkers package in Seurat. The resulting clusters and their corresponding gene markers were visualized via the ggplot2 package.

For the analysis of downstream genes of NRF2, raw data were obtained from the GEO database, comprising transcriptome profiles of wild‐type and NRF2 knockout RAW264.7 cells treated with LPS and IFN‐γ (GSE71695 and GSE71263).^[^ ^47^, ^48^ ^]^ Protein‐protein interaction networks involving NRF2 were acquired from the STRING database (https://string‐db.org/). All bioinformatics analyses were also performed using R software. Sequence read quality was assessed using FastQC, followed by read count normalization and differential expression analysis using the DESeq2 package. Hierarchical clustering of differentially expressed genes was performed using a heatmap, while comparative analysis of gene sets between experimental conditions was visualized using VennDiagram.

### Cell Culture

The murine macrophage cell line RAW264.7 was purchased from Procell Life Science & Technology (Wuhan, China). The RAW264.7 cells were thawed rapidly in a 37 °C water bath and cultured in Dulbecco's Modified Eagle Medium (DMEM; Gibco, USA) supplemented with 10% fetal bovine serum (FBS; Gibco, USA) and 1% penicillin‐streptomycin (HyClone, USA) at 37 °C in a humidified 5% CO_2_ atmosphere. Cells were maintained in the exponential growth phase and passaged at ≈80% confluence. For stimulation experiments, cells were seeded in appropriate culture dishes and allowed to reach ≈60% confluence. Before stimulation, cells were washed twice with PBS and maintained in serum‐free DMEM for 2 h to synchronize cell cycle progression. Cell activation was achieved by treatment with 200 ng mL^−1^ ultrapure LPS (tlrl‐3pelps, InvivoGen, USA) and 40 ng mL^−1^ recombinant murine IFN‐γ (315‐05, PeproTech, USA) for 6 h. Following stimulation, cells were harvested by brief trypsinization (0.25% trypsin‐EDTA, 37 °C, 3 min) and immediately neutralized with a complete medium. Cell suspensions were washed twice with ice‐cold PBS and processed for RNA extraction, protein isolation, and ROS detection.

### Cell Uptakes

Well‐maintained RAW264.7 cells were seeded in glass‐bottom confocal dishes (Corning, USA). Upon reaching 60% confluence, cells were washed twice with sterile PBS and maintained in serum‐free DMEM for 2 h to synchronize cell cycles before treatment. Transfection complexes were prepared by incubating Lipofectamine 3000 (Invitrogen, USA) with respective nucleic acids (siRNA, ssDNA, tFNA, or DDN) in Opti‐MEM reduced serum medium for 15 min at room temperature, following the manufacturer's protocol. Treated cells were incubated for specified durations (1, 3, 6, or 12 h) under standard culture conditions. Following incubation, cells were gently washed three times with pre‐warmed PBS and prepared for subsequent immunofluorescence staining procedures.

### RNA Sequencing (RNA‐seq)

The extraction of total RNA followed the standard protocol. The mRNA was reverse‐transcribed into complementary DNA (cDNA). The cDNA was end‐repaired, adenylated, and ligated to Illumina sequencing adapters. These prepared libraries were sequenced by Gene Denovo Biotechnology Co. (Guangzhou, China), generating FASTQ‐formatted data. FASTQ files were subjected to quality control via FastQC software. The filtered reads were subsequently aligned to a reference genome to restore their original genomic positions. The raw data were submitted on the NCBI (SUB14905858).

### Western Blot (WB)

SDS‐PAGE was performed using a commercially available gel preparation system (Beyotime, China). The separating gel (10% acrylamide) was prepared by mixing components A and B at a 1:1 ratio (v/v) and allowed to polymerize for 30 min between pre‐cleaned glass plates. Subsequently, a 5% stacking gel was layered atop the polymerized separating gel and allowed to cure for an additional 20 min. Protein samples (7 µL, normalized to 2 µg µL^−1^ by BCA protein assay) were loaded alongside Precision Plus Protein Dual Color standards (Bio‐Rad, 5 µL/lane). Electrophoresis was conducted under 60 V constant voltage for 30 min and 120 V constant voltage until the dye front reached the gel bottom. Following electrophoresis, gels were equilibrated in transfer buffer for 15 min. Proteins were electrotransferred to 0.45 µm PVDF membranes (Millipore, USA) at 70 V for 60 min using a Trans‐Blot SD semi‐dry transfer cell (Bio‐Rad, USA). Membranes were blocked with 5% non‐fat dry milk in TBST for 60 min at room temperature with gentle agitation. Primary antibodies (Table S5, Supporting Information) were diluted in blocking buffer according to manufacturer specifications and incubated overnight at 4 °C with continuous shaking. After three 10‐min washes with TBST, membranes were incubated with HRP‐conjugated secondary antibodies for 60 min at room temperature. Protein bands were visualized using an enhanced chemiluminescence substrate (Beyotime, China) and imaged using Imaging System. Densitometric analysis was performed using ImageJ software. All experiments included three biological replicates, with representative blots selected for presentation.

### Quantitative Real Time Polymerase Chain Reaction (qRT‐PCR)

Total RNA was extracted from ≈20 mg of fresh liver tissue using a commercial RNA isolation kit (Beyotime, China) according to the manufacturer's protocol. Briefly, tissue samples were homogenized in 250 µL Buffer RL1. The homogenate was incubated at room temperature for 30 min to ensure complete lysis. Flow‐through was mixed with 400 µL Buffer RL2 and loaded onto RNA‐only columns. RNA was eluted with 30 µL RNase‐free water preheated to 65 °C and incubated for 5 min at room temperature. RNA concentration was determined using a NanoDrop 2000 spectrophotometer (Thermofisher, USA). All samples were normalized to 1 µg µL^−1^ with RNase‐free water.

For each reaction, 1 µg total RNA was treated with 4× gDNA wiper Mix in a 20 µL reaction volume. The mixture was incubated at 42 °C for 2 min to remove contaminating genomic DNA. Reverse transcription was performed using 4× RT PCR SuperMix (5 µL per reaction) under the following conditions: 37 °C for 15 min and 85 °C for 5 s. qPCR reactions were prepared in 20 µL volumes containing cDNA template, GeneFast SYBR Green qPCR Mix (Beyotime, China), primers, and nuclease‐free water. Primers specific to the target genes were used, and their sequences were listed in Table S6 (Supporting Information). The reaction was performed on a CFX96 instrument (Bio‐rad, USA) using FastStart Universal SYBR Green Master Mix (Beyotime, China) according to the manufacturer's instructions. Fold changes were determined using the arithmetic comparative method (2 ^−ΔΔCt^).

### Immunofluorescence

Tissue sections were subjected to standardized antigen retrieval procedures to optimize epitope exposure. Briefly, sections were immersed in preheated citrate‐based antigen retrieval buffer (pH 6.0) and repaired via thermal protocol. Sections were incubated with blocking solution consisting of 1% donkey serum (Beyotime, China) containing 0.3% Triton X‐100. Primary antibodies, as specified in Table S5 (Supporting Information), were diluted in PBS and applied to sections overnight at 4 °C. Following three sequential 5‐min washes with PBS, sections were incubated with secondary antibodies for 60 min at room temperature. Tissue sections were counterstained with 4′,6‐diamidino‐2‐phenylindole (DAPI; Beyotime, China) for precise nuclear localization. High‐resolution images were obtained using a confocal laser microscope (Leica, Germany). Three representative fields of view were systematically captured per section.

### Primary Murine Hepatic NPCs Isolation

C57BL/6J mice were anesthetized, and the portal vein was cannulated with a 24‐gauge polyethylene catheter. Perfusion with calcium‐free Hank's Balanced Salt Solution supplemented with 0.5 mm EGTA for 5 min to remove circulating blood cells. Perfusion with HBSS containing 0.8 mg mL^−1^ collagenase IV and 5 mm calcium chloride for 10 min until complete tissue digestion was visually confirmed. The hepatic capsule was gently disrupted, and the cell suspension was agitated to ensure complete dissociation. NPCs were collected by centrifugation of the supernatant at 300 × g for 10 min at 4 °C.

### FCM

Isolated NPCs were aliquoted into pre‐chilled 5 mL polystyrene round‐bottom flow cytometry tubes (Corning, USA) at a density of 1 × 10⁶ cells per tube in 100 µL volume. Cells were incubated with anti‐mouse CD16/32 Fc block (BioLegend, USA) in Cell Staining Buffer. Fluorochrome‐conjugated antibodies (Table S7, Supporting Information) were titrated to optimal concentrations and added to cell suspensions. Samples were acquired within 2 h of staining using a flow cytometer (BD, USA).

### ROS Detection

DHE Fluorescence Microscopy Assay: Cells or tissue sections cultured on confocal dishes were incubated with 5 µm DHE (Beyotime, China) in serum‐free medium at 37 °C for 15 min protected from light. Following incubation, samples were washed three times with PBS. High‐resolution images were obtained using a confocal laser microscope.

Flow Cytometric ROS Detection: Cells cultured were incubated with 10 µm 2′,7′‐dichlorodihydrofluorescein diacetate (Beyotime, China) in serum‐free medium at 37 °C for 20 min. Cells were trypsinized and washed twice with ice‐cold PBS. Samples were acquired within 2 h of staining using flow cytometer.

### Statistics

Statistical analysis was conducted by GraphPad Prism software (San Diego, USA). The comparison between the 2 groups was performed using Student's *t* test. Comparisons among multiple groups were conducted using one‐way ANOVA. Pairwise comparisons between multiple groups were analyzed using the q‐test. The correlation analysis between the 2 groups was assessed using Spearman's rank correlation analysis. Two‐tailed *P* values less than 0.05 were considered statistically significant.

## Conflict of Interest

All the authors declare that they have no conflicts of interest.

## Author Contributions

B.W. and M.P. designed the research; B.W., M.P., J.X., and Y.H. performed the research and analyzed the data; B.W. and M.P. wrote the manuscript; F.F., Y.L., and C. provided assistance during data collection and analysis.

## Supporting information



Supporting Information

## Data Availability

The data that support the findings of this study are available on request from the corresponding author.
