# Supplementary material for: A Dynamic DNA Nano‐Antioxidant Targeting Galectin‐3 Attenuates Liver Fibrosis via Reducing Macrophage Oxidative Stress
Source: Adv Sci (Weinh). 2025 Oct 30;13(4):e09977. doi: 10.1002/advs.202509977 (PMC12822432; doi:10.1002/advs.202509977)
Supplement: Supplementary file 1 — Supporting Information [file ADVS-13-e09977-s001.docx]

**Supplementary information**

**A Dynamic DNA Nano-antioxidant Targeting Galectin-3 Attenuates Liver Fibrosis via Reducing Macrophage Oxidative Stress**

Mengjia Peng^1,2,3^, Jianguo Xu^1^, Youjian Hong^1^, Fei Fang^1^, Yan Li^4^, Ciduo^1^, Bowen Wang*^1,2,3^.

1, Department of Emergency, the General Hospital of Tibet Military Command, Lhasa, China;

2, Department of Gastroenterology, Laboratory of Gastroenterology and Hepatology, West China Hospital, Sichuan University, Chengdu, China;

3, State Key Laboratory of Oral Diseases, National Center for Stomatology, National Clinical Research Center for Oral Diseases, West China Hospital of Stomatology, Sichuan University, Chengdu, China;

4, Physical Examination Center, General Hospital of Western Theater Command, Chengdu, China.

***Correspondence:**

Professor Bowen Wang, Ph.D., M.D., E-mail: [1491867642@qq.com](mailto:1491867642@qq.com).

**
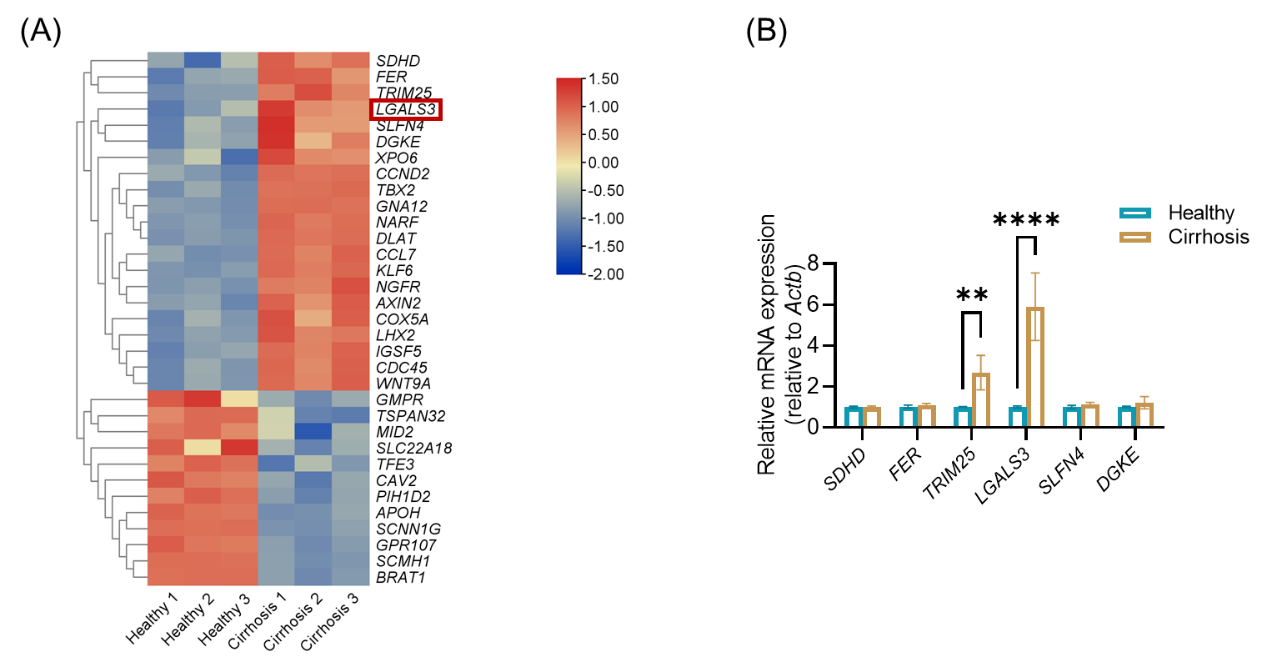
**

**Supplementary figure 1.** (A) Heatmaps of differentially expressed genes in healthy controls and cirrhotic patients (n = 3). (B) The expression of *SDHD*, *FER*, *TRIM25*, *LGALS3*, *SLFN4*, and *DGKE* in both healthy and cirrhosis groups (n=3).


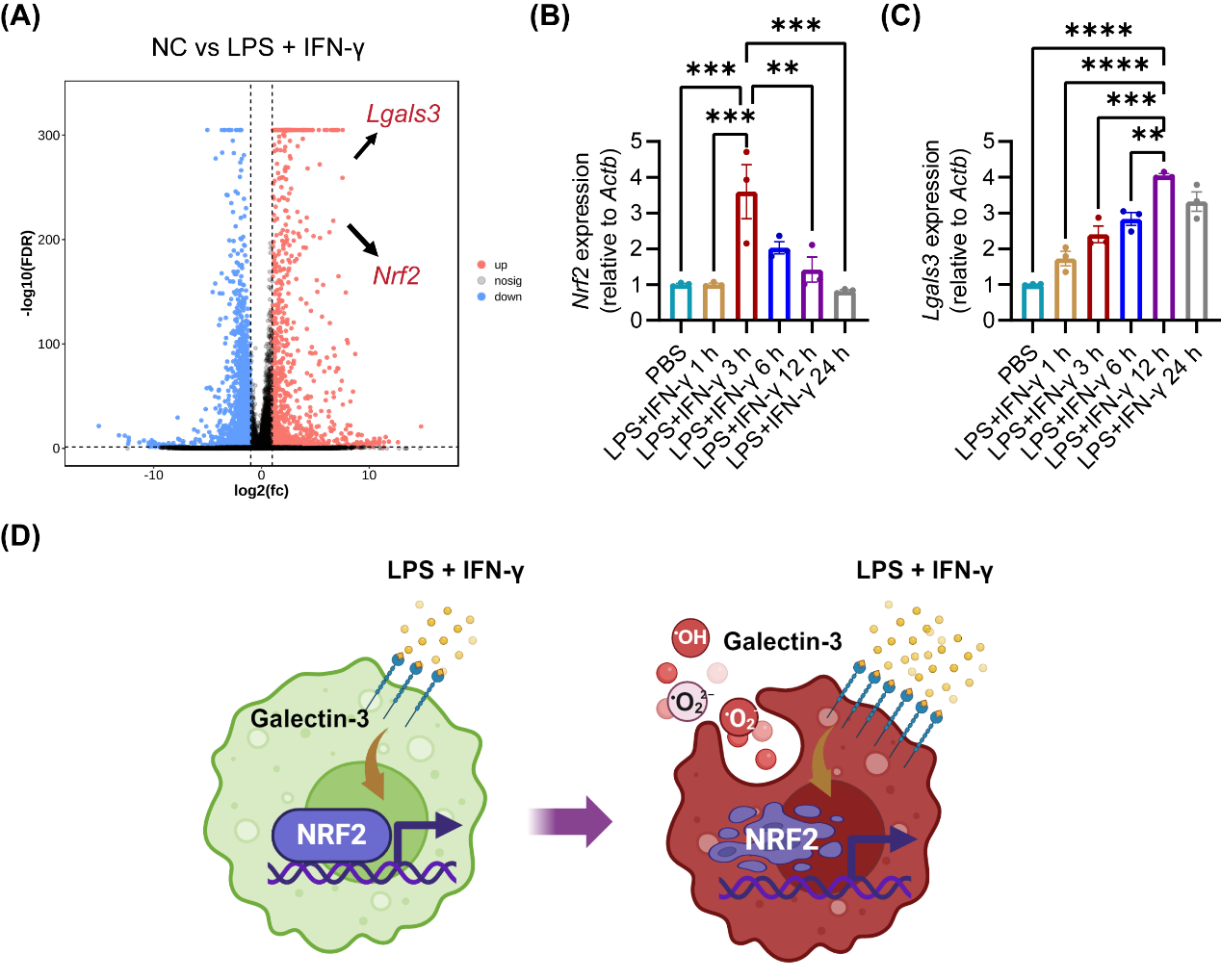


**Supplementary figure 2.** LPS and IFN-γ depleted the NRF2 via promoting Galectin-3 expression.

(A) RAW264.7 cells were treated with PBS or LPS and IFN-γ. The RAW264.7 cells were collected and performed to RNA-seq. Volcano plots of differentially expressed genes between the groups; The expression of *Lgals3* (B) and *Nrf2* (C) in the groups. Data are presented as the mean ± SD (n = 3). ***P* < 0.01, ****P* < 0.001, *****P* < 0.0001. (D) Schematic illustration of LPS and IFN-γ induced macrophage oxidative stress and ROS release through Galectin-3 promotion and subsequent leading to NRF2 depletion.


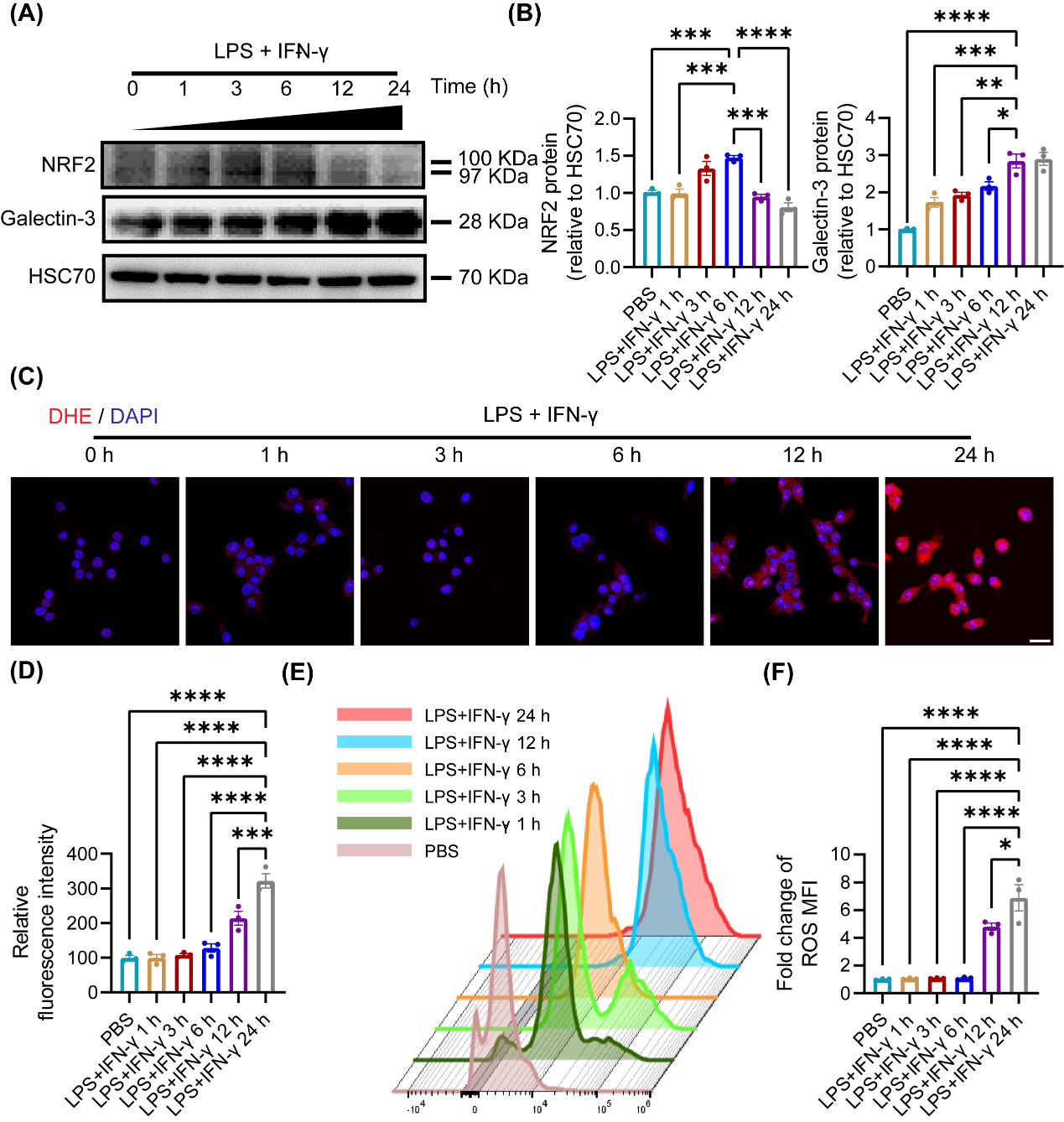


**Supplementary figure 3.** LPS and IFN-γ promoted the oxidative stress and ROS release in macrophages.

(A) RAW264.7 cells were treated with PBS or BiRDS, then stimulated with LPS + IFN-γ by different time points. Representative western blotting images of NRF2 and Galectin-3 expression in each group; (B) Statistic analysis of (A), Data are presented as the mean ± SD (n = 3). **P* < 0.05, ***P* < 0.01, ****P* < 0.001, *****P* < 0.0001; (C) Representative immunofluorescence images of DHE and DAPI in each group, scale bar = 25 μm; (D) Statistic analysis of (C), Data are presented as the mean ± SD (n = 3). ****P* < 0.001, *****P* < 0.0001; (E) Representative flow cytometry images of ROS in each group; (F) Statistic analysis of (E), Data are presented as the mean ± SD (n = 3). **P* < 0.05, *****P* < 0.0001.


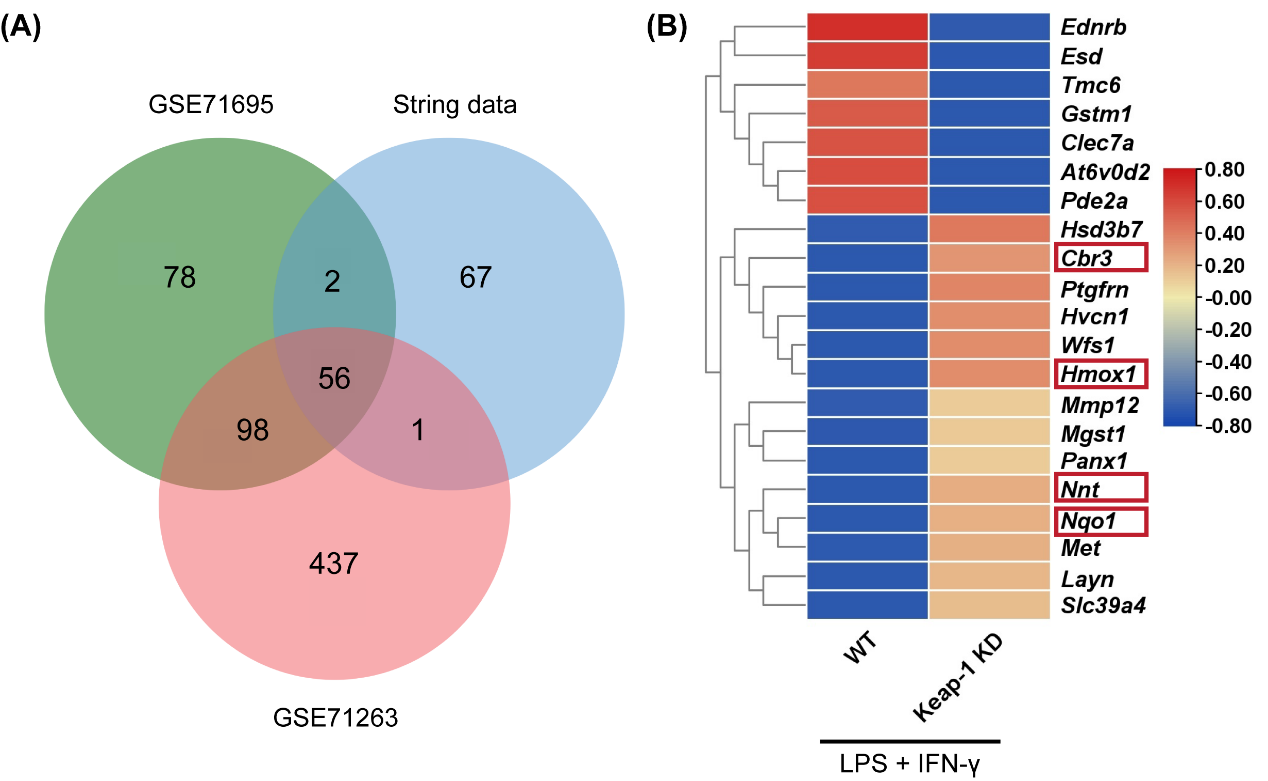


**Supplementary figure 4.** Screening the downstream genes of *Nrf2*.

(A) Venn diagram of differentially expressed genes among the datasets of GSE71695, GSE71263, and String data; (B) Heatmaps of the top differentially expressed genes of the 56 genes screening by (A).


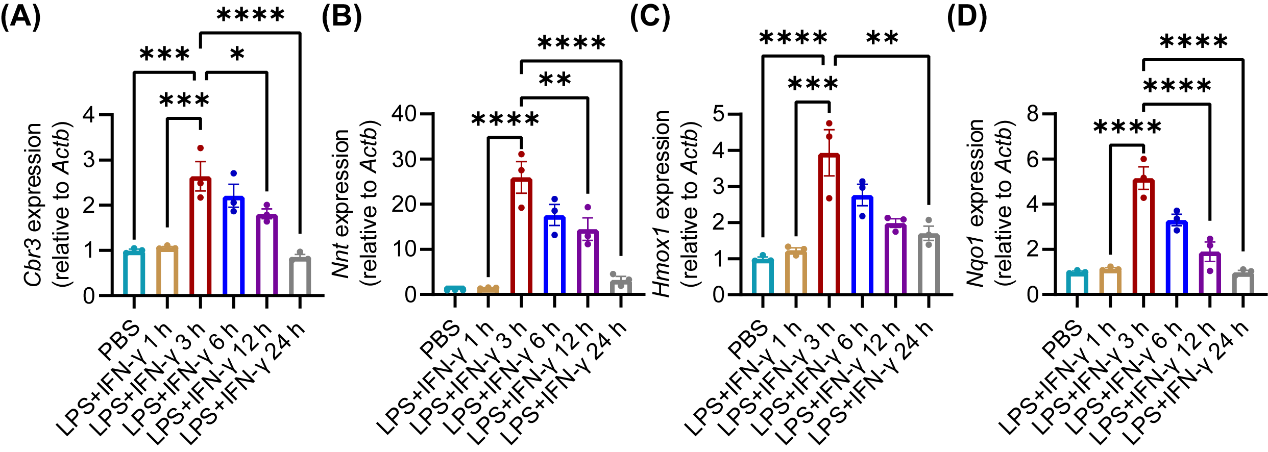


**Supplementary figure 5.** RAW264.7 cells were treated with PBS or LPS + IFN-γ by different time points. The expression of *Cbr3* (A), *Nnt2* (B), *Hmox1* (C) and *Nqo-1* (D) in each group.


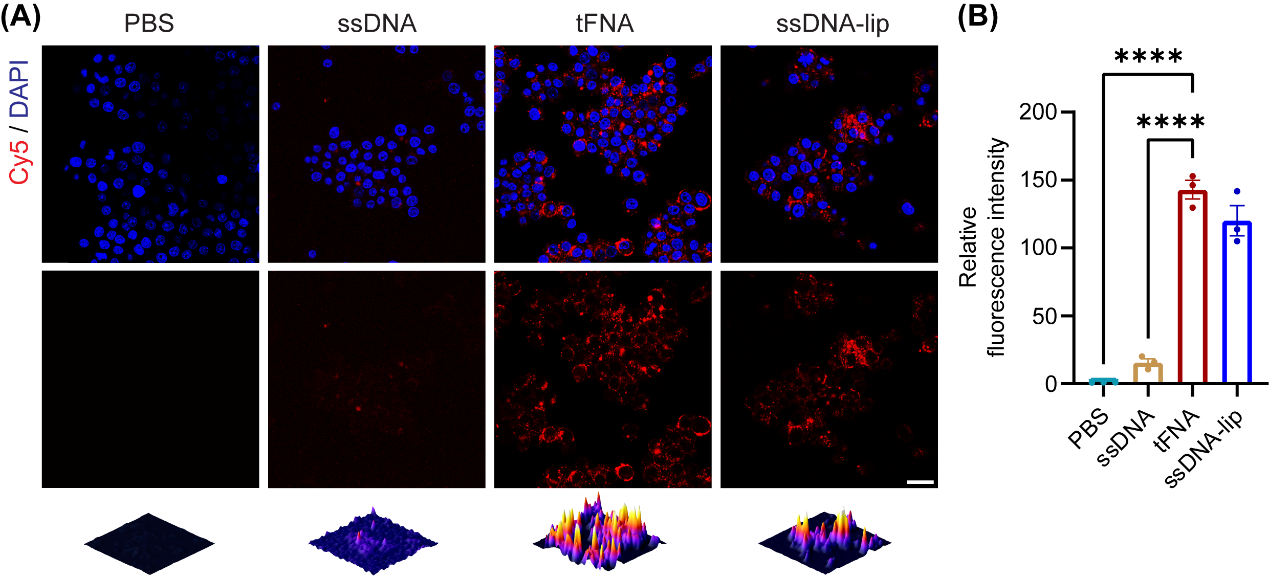


**Supplementary figure 6.** Cell Uptakes ability of tFNA in RAW264.7.

(A) The fluorescence images of RAW264.7 cells that treated with PBS, Cy5-ssDNA, Cy5-tFNA, and Cy5-ssDNA-lip at 6 hours. Scale bar = 25 μm; (B) Statistic analysis of (A). Data are presented as the mean ± SD (n = 3). *****P* < 0.0001.

**Supplementary figure 7.** RAW264.7 cells were treated with PBS or DNN, then stimulated with LPS + IFN-γ by different time points. The expression of *Nqo1* in each group.


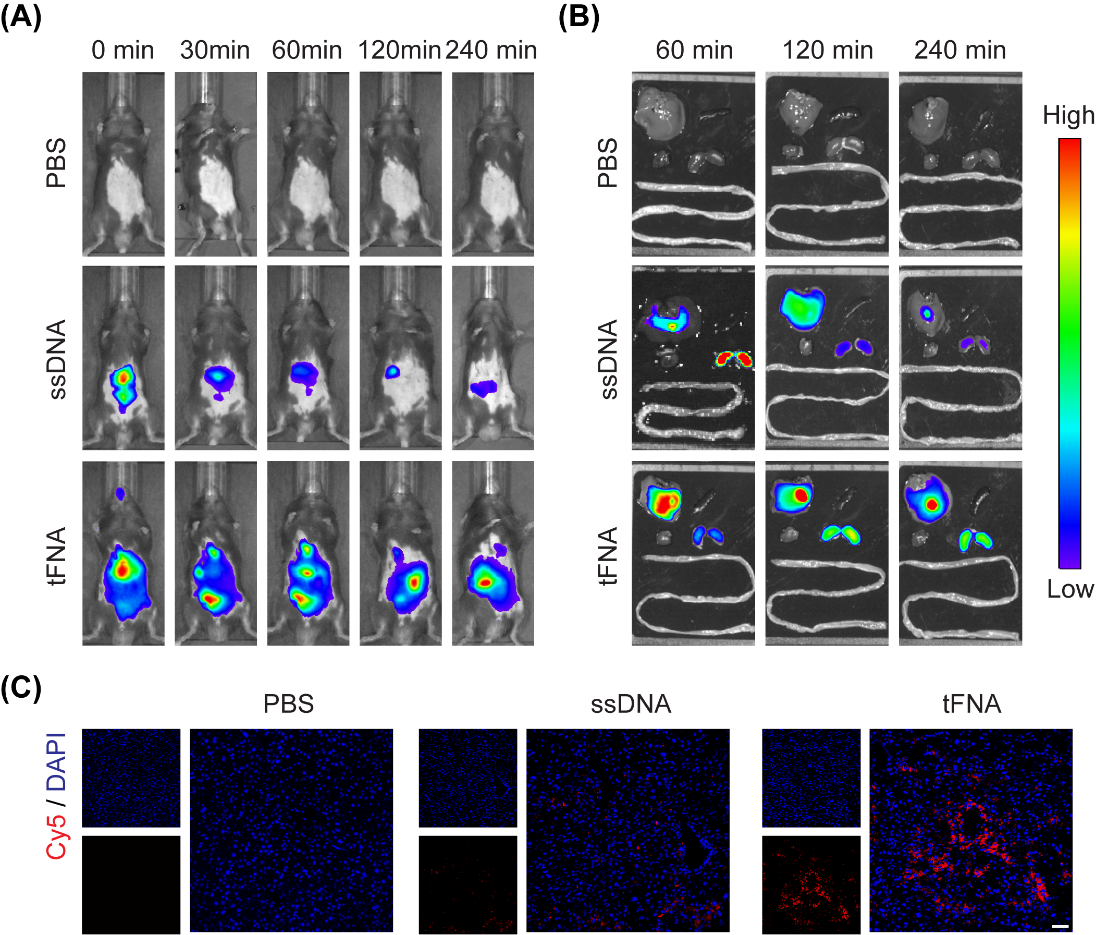


**Supplementary figure 8.** Liver-specific delivery of tFNA.

(A) *In vivo* fluorescence images of the whole body were taken at different time points after i.p. administration of PBS, Cy5-ssDNA, and Cy5-tFNA. (B) *In vivo* fluorescence images of the isolated organs (liver, spleen, heart, kidney, and intestine) were taken at different time points after i.p. administration of PBS, Cy5-ssDNA, and Cy5-tFNA. (C) Representative immunofluorescence images of Cy5 and DAPI in liver, scale bar = 50 μm.


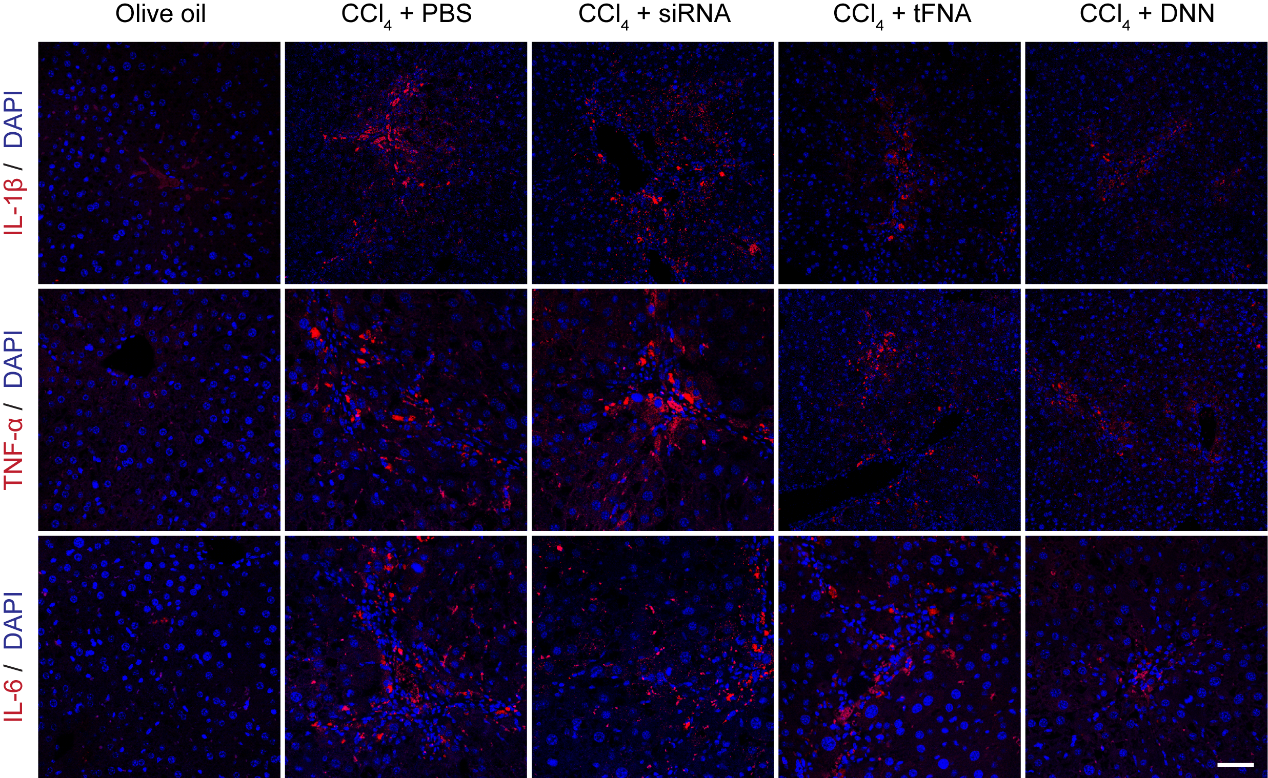


**Supplementary figure 9.** Representative immunofluorescence images of IL-1β, TNF-α, IL-6, and DAPI in each group, scale bar = 25 μm.


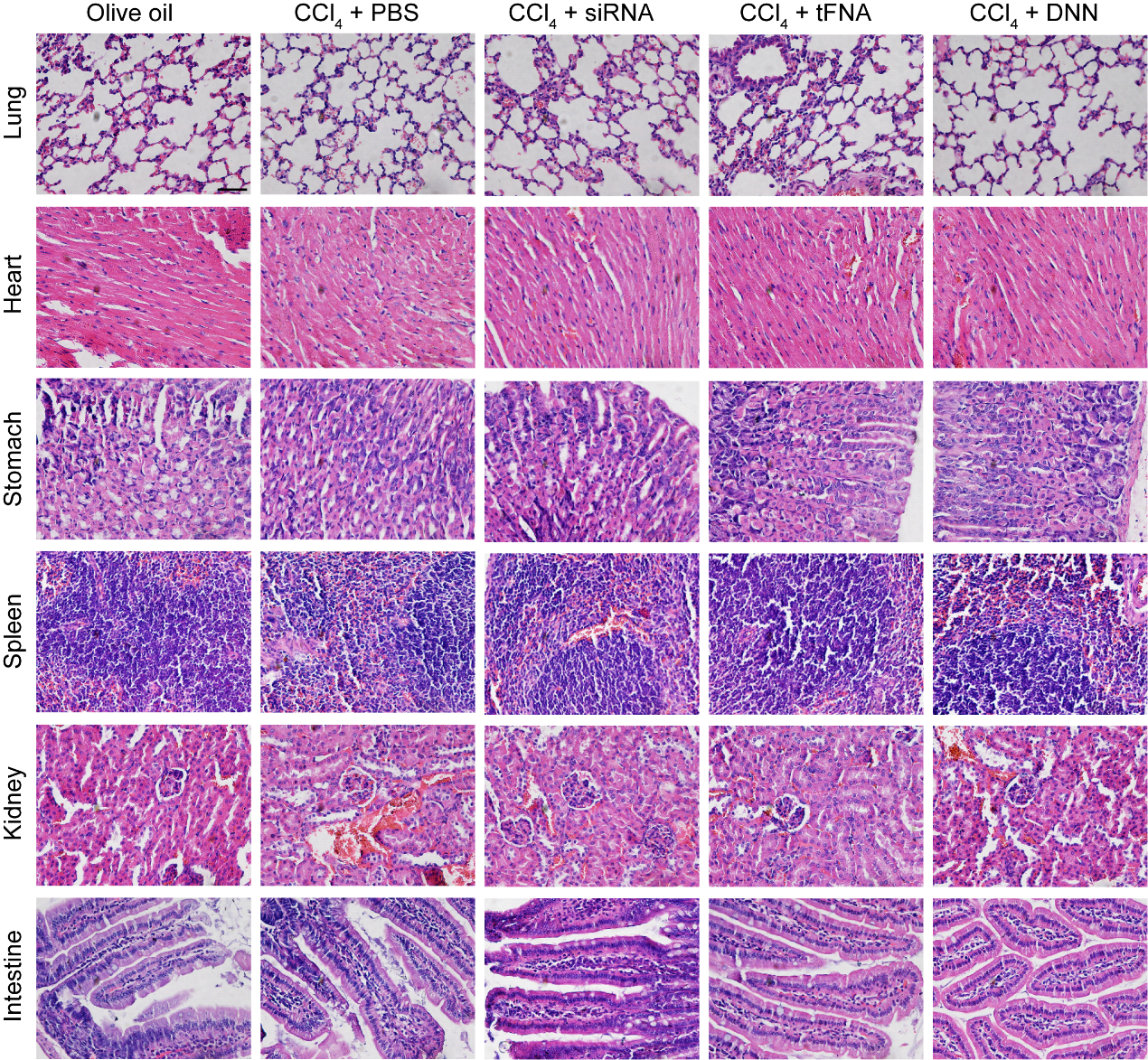


**Supplementary figure 10.** Representative H&E staining images of intestine, lungs, spleen, kidneys, stomach, and heart of different groups. Scale bar = 100 μm.

**Supplementary table 1. List of sequences for tFNA**

| **Name** | **Sequence** |
| --- | --- |
| **Sequences for tFNA** | |
| **S1** | \| ATTTATCACCCGCCATAGTAGACGTATCACCAGGCAGTTGAGACGAACATTCCTAAGTCTGAA \|  \| \| --- \| --- \| |
| **S2** | ACATGCGAGGGTCCAATACCGACGATTACAGCTTGCTACACGATTCAGACTTAGGAATGTTCG |
| **S3** | ACTACTATGGCGGGTGATAAAACGTGTAGCAAGCTGTAATCGACGGGAAGAGCATGCCCATCC |
| **S4** | ACGGTATTGGACCCTCGCATGACTCAACTGCCTGGTGATACGAGGATGGGCATGCTCTTCCCG |
| **Cy5-S1** | Cy5-ATTTATCACCCGCCATAGTAGACGTATCACCAGGCAGTTGAGACGAACATTCCTAAGTCTGAA |
| **Cy5-S2** | Cy5-ACATGCGAGGGTCCAATACCGACGATTACAGCTTGCTACACGATTCAGACTTAGGAATGTTCG |
| **Cy5-S3** | Cy5- ACTACTATGGCGGGTGATAAAACGTGTAGCAAGCTGTAATCGACGGGAAGAGCATGCCCATCC |

**Supplementary table 2. List of sequences for siRNA**

| **Sequences for siRNA of Galectin-3** | |
| --- | --- |
| **siRNA1** | Sense: CGCAAACAGGAUUGUUCUATT  Anti-sence: UGAUGGCAACAAUAUCACTT |
| **siRNA2** | Sense: CGAGAGUCAUUGUGUGUAATT  Anti-sense: UUACACACAAUGACUCUCCTT |
| **siRNA3** | Sense: GGUCAACGAUGCUCACCUATT  Anti-sense: UAGGUGAGCAUCGUUGACCTT |

The underlined sequence is a ribonucleotide.

**Supplementary table 3. List of sequences for DNN**

| **Sequences for DNN** | |
| --- | --- |
| **DNN-SH1** | UAGAACAAAGGATGGGCATGCTCTTCCCGACGGTATTGGACCCTCGCATGAUGUUUGCGTAAG |
| **DNN-SH2** | UAGAACAAACATGCGAGGGTCCAATACCGACGATTACAGCTTGCTACACGAUGUUUGCGTAAG |
| **DNN-SH3** | UAGAACAAACGTGTAGCAAGCTGTAATCGACGGGAAGAGCATGCCCATCCAUGUUUGCGTAAG |
| **siRNA** | CGCAAACAGGAUUGUUCUA |
| **Cy5-siRNA** | Cy5-CUUACGCAAACAGGAUUGUUCUA |

The underlined sequence is a ribonucleotide.

**Supplementary table 4. Clinical information of patients with cirrhosis and healthy controls**

| Group | Age | Gender | Diagnosis | HBV | Sample |
| --- | --- | --- | --- | --- | --- |
| Healthy 1 | 57 | F | Normal | - | Liver tissue |
| Healthy 2 | 26 | F | Normal | - | Liver tissue |
| Healthy 3 | 42 | M | Normal | - | Liver tissue |
| Healthy 4 | 69 | F | Normal | - | Liver tissue |
| Healthy 5 | 51 | F | Normal | - | Liver tissue |
| Healthy 6 | 49 | F | Normal | - | Liver tissue |
| Healthy 7 | 23 | M | Normal | - | Liver tissue |
| Healthy 8 | 51 | F | Normal | - | Liver tissue |
| Healthy 9 | 39 | M | Normal | - | Liver tissue |
| Healthy 10 | 46 | F | Normal | - | Liver tissue |
| Healthy 11 | 44 | F | Normal | - | Liver tissue |
| Cirrhosis 1 | 79 | M | Cirrhosis | - | Liver tissue |
| Cirrhosis 2 | 54 | M | Cirrhosis | + | Liver tissue |
| Cirrhosis 3 | 70 | M | Cirrhosis | + | Liver tissue |
| Cirrhosis 4 | 57 | M | Cirrhosis | + | Liver tissue |
| Cirrhosis 5 | 39 | M | Cirrhosis | - | Liver tissue |
| Cirrhosis 6 | 56 | M | Cirrhosis | - | Liver tissue |
| Cirrhosis 7 | 50 | M | Cirrhosis | + | Liver tissue |
| Cirrhosis 8 | 58 | M | Cirrhosis | + | Liver tissue |
| Cirrhosis 9 | 53 | M | Cirrhosis | - | Liver tissue |
| Cirrhosis 10 | 60 | M | Cirrhosis | - | Liver tissue |
| Cirrhosis 11 | 62 | F | Cirrhosis | + | Liver tissue |
| Cirrhosis 12 | 67 | M | Cirrhosis | + | Liver tissue |
| Cirrhosis 13 | 66 | M | Cirrhosis | - | Liver tissue |
| Cirrhosis 14 | 59 | M | Cirrhosis | - | Liver tissue |
| Cirrhosis 15 | 61 | M | Cirrhosis | + | Liver tissue |
| Cirrhosis 16 | 57 | M | Cirrhosis | - | Liver tissue |

**Supplementary table 5. Antibody list for Immunofluorescence and Western blotting**

| **Antibody** | **Application** | **Dilution** | **Source** | **Company and cat no** |
| --- | --- | --- | --- | --- |
| **αSMA** | IF  WB | 1:200  1:500 | Rabbit | Abcam # ab124964 |
| **Collagen 1** | IF  WB | 1:100  1:1000 | Goat  Rabbit | Southern Biotech #1310-01  Abcam #260043 |
| **F4/80** | IF | 1:100 | Rabbit | Cell Signaling Technology #70076 |
| **Galectin-3** | IF  WB | 1:100  1:1000 | Rabbit | Cell Signaling Technology #89572 |
| **GAPDH** | WB | 1:10000 | Mouse | ABclonal #AC033 |
| **HSC70** | WB | 1:10000 | Mouse | Santa Cruz Biotechnology #Sc-7298 |
| **IL-1β** | IF | 1:200 | Rabbit | Abcam # ab234437 |
| **IL-6** | IF | 1:200 | Rabbit | Abcam # ab290735 |
| **NRF2** | WB | 1:1000 | Rabbit | Cell Signaling Technology #12721 |
| **4HNE** | WB | 1:1000 | Rabbit | Abcam # ab46545 |
| **TNF-α** | IF | 1:5000 | Rabbit | Abcam # ab183218 |

αSMA: α-Smooth Muscle Actin; GAPDH: Glyceraldehyde-3-phosphate dehydrogenase; HSC70: Heat shock cognate protein 70; IF: Immunofluorescence; IL-1β: interleukin 1 beta; IL-6: Interleukin 6; NRF2: Nuclear factor erythroid 2-related factor 2; 4HNE: 4-Hydroxynonenal; TNF-α: Tumor necrsis factor α; WB: Western blot.

**Supplementary table 6. List of primers for qRT‒PCR**

| **Gene** | **Sequence-forward** | **Sequence-reverse** |
| --- | --- | --- |
| ***Actb*** | TGACGTTGACATCCGTAAAG | GAGGAGCAATGATCTTGATCT |
| ***Cbr3*** | GTCTGCACTGAGTTACTGCCTATA | CAACCTTCCCTCTCATGGACTTC |
| ***Dgke*** | AAGTTGGCACCATGGAAGCG | CCCAGAGGCAAAACTGCAAC |
| ***Fer*** | CACACCCTCGAATAATGACGC | ATTCTGATGGAGCTGTGCCC |
| ***Hmox1*** | CCTCACAGATGGCGTCACTT | GCTGATCTGGGGTTTCCCTC |
| ***Il1b*** | TCTTTGAAGTTGACGGACCC | TGAGTGATACTGCCTGCCTG |
| ***Il6*** | GAAAGTGGCTATGCAGTTTGAA | GAGGTAAGCCTACACTTTCCAAGA |
| ***Lgals3*** | CTTCTGGACAGCCAAGTGC | AGGCAGGTTATAAGGCACAATC |
| ***Nnt*** | CGCGAAAGAGATGTCCAAAGAGT | CCACCGTAGAGAGCAGCTAAATA |
| ***Nqo1*** | AGTGGCATCCTGCGTTTCTG | TAGAGTGGGGTCTCCTCCCA |
| ***Nrf2*** | CCATTTACGGAGACCCACCG | TGGGATTCACGCATAGGAGC |
| ***Sdhd*** | ACACCCTCGAATAATGACGCA | AACGGGTCTGCTCTTCAGTG |
| ***Slfn4*** | ACTCTGAAGGCTTGGGAAACA | GTCCTTACTCTGCCCCGAAC |
| ***Tnfa*** | CCACATCTCCCTCCAGAAAA | AGGGTCTGGGCCATAGAACT |
| ***Trim25*** | CAACTGTGACCACGGCTTTG | AGCCTTCAGATCCAAGTGGC |

*Actb*: Homo sapiens actin beta; *Cbr3*: Carbonyl reductase 3; *Dgke*: Diacylglycerol kinase epsilon; *Fer*: Tyrosine kinase; *Hmox1*: Heme oxygenase (decycling) 1; *Il1b*: interleukin 1 beta; *Il6*: Interleukin 6; *Lgals3*: Lectin, galactoside-binding, soluble, 3; *Nnt*: Nicotianamine aminotransferase; *Nqo-1*: NAD(P)H quinone oxidoreductase 1; *Nrf2*: Nuclear factor erythroid 2-related factor 2; *Sdhd*: Succinate dehydrogenase complex subunit D; *Slfn4*: Schlafen 4; *Tnfa*: Tumor necrsis factor α; Trim25: tripartite motif containing 25.

**Supplementary table 7. Antibody list for Flow cytometry**

| **Antibody** | **Format** | **Dilution** | | **Source** | | **Company and cat no** | |
| --- | --- | --- | --- | --- | --- | --- | --- |
| **CD45** | Alexa Fluor 700 | 1:200 | Rat | | Biolegend #103128 | |  |
| **F4/80** | FITC | 1:200 | Rat | | Biolegend #123108 | |  |
| **CD11b** | APC | 1:200 | Rat | | Biolegend #101212 | |  |
| **CD86** | PE | 1:200 | Rat | | Biolegend #159204 | |  |

FITC: Fluorescein Isothiocyanate; APC: Allophycocyanin; PE: Phycoerythrin
